# EGFR‐Targeting IgG1 Antibody Enhances NK Cell‐Mediated Tumor Killing in KRAS‐Mutant Pancreatic Cancer

**DOI:** 10.1002/mco2.70860

**Published:** 2026-06-23

**Authors:** Ruoxi Xiao, Xiaoxiao Li, Ping Li, Junjin Wang, Xiaoyuan Sun, Chenyang Zhao, Zimin Liu, Ruining Gong, Minghan Ren, Ke Lei, He Ren

**Affiliations:** ^1^ Shandong Provincial Key Laboratory of Clinical Research for Pancreatic Diseases Tumor Immunology and Cytotherapy Medical Research Center The Affiliated Hospital of Qingdao University Qingdao China; ^2^ Department of Clinical Medicine Qingdao University Qingdao China; ^3^ Gastrointestinal Cancer Institute/Pancreatic Disease Institute The Affiliated Hospital of Qingdao University Qingdao China; ^4^ Cheeloo College of Medicine Qilu Hospital (Qingdao) Shandong University Qingdao China; ^5^ Tumor Immunology and Cytotherapy Medical Research Center The Affiliated Hospital of Qingdao University Qingdao China

**Keywords:** antibody‐dependent cellular cytotoxicity, EGFR, NK cells, pancreatic ductal adenocarcinoma

## Abstract

KRAS‐mutant pancreatic ductal adenocarcinoma (PDAC) exhibits intrinsic resistance to epidermal growth factor receptor (EGFR)‐targeted therapies owing to constitutive downstream pathway activation. Nevertheless, IgG1 antibodies may retain therapeutic activity through natural killer (NK) cell‐mediated antibody‐dependent cellular cytotoxicity (ADCC), thereby bypassing EGFR downstream signaling. However, whether EGFR‐targeted IgG1 antibody‐mediated ADCC remains effective in KRAS‐mutant PDAC, and what determines therapeutic responsiveness, remain unclear. Here, we investigated whether nimotuzumab‐mediated ADCC remains effective despite oncogenic KRAS signaling and explored the determinants of its therapeutic efficacy. Using complementary in vitro and in vivo models, including PDAC cell–NK cell co‐culture systems, 3D tumor spheroids, and immunodeficient mouse models (subcutaneous and circulating tumor cell‐derived xenografts), we demonstrated that combined nimotuzumab and adoptive NK cell therapy exerts potent antitumor efficacy in PDAC. Mechanistically, this treatment drives robust NK cell functional activation (CD107a/IFN‐γ/TNF‐α), enhances tumor homing, and induces immunogenic cell death. Collectively, our findings demonstrate that KRAS mutations do not compromise nimotuzumab‐mediated ADCC, whereas tumor EGFR expression serves as a predictor of therapeutic responsiveness. Ultimately, this study establishes EGFR‐directed NK cell immunotherapy as a promising therapeutic strategy for KRAS‐mutant PDAC and provides a rationale for integrating targeted antibodies with cellular immunotherapies in other EGFR‐expressing malignancies.

## Graphical abstract

In KRAS‐mutant pancreatic cancer, persistent downstream signaling can limit the efficacy of EGFR‐targeting mAbs. However, the addition of NK cells enables nimotuzumab to effectively direct a potent antitumor response via antibody‐dependent cellular cytotoxicity (ADCC), overcoming this resistance mechanism.

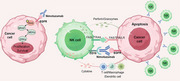

## 1 Introduction

Pancreatic ductal adenocarcinoma (PDAC) represents a therapeutic challenge in oncology, with 5‐year survival rates stagnating below 15% despite decades of research [1]. This dismal prognosis results from aggressive tumor biology characterized by delayed diagnosis, metastatic propensity, and profound therapy resistance [2, 3, 4, 5]. Current treatment options provide only limited survival benefits [6], while the highly immunosuppressive tumor microenvironment renders most patients unresponsive to immune checkpoint blockade [7, 8, 9]. These challenges underscore the urgent need for novel immunotherapeutic strategies for PDAC.

Anti‐epidermal growth factor receptor (EGFR) monoclonal antibodies (mAbs) have achieved remarkable success in multiple cancers [10, 11]; however, they show limited efficacy in PDAC despite high EGFR expression. This diminished response is closely associated with near‐universal KRAS mutations [12] that constitutively activate the MAPK/PI3K pathway, sustaining proliferation independently of upstream EGFR signaling [13]. Clinical evidence consistently supports this molecular rationale: EGFR‐targeting regimens demonstrate benefits only in wild‐type KRAS subgroups, with minimal efficacy in unselected PDAC populations [14, 15, 16, 17]. IgG1 mAbs possess a bifunctional architecture: the Fab domain specifically binds to target antigens, whereas the Fc domain engages Fcγ receptors (FcγRs) to initiate potent effector functions [18]. Through FcγRs engagement, IgG1 antibodies can trigger multiple effector mechanisms, among which antibody‐dependent cellular cytotoxicity (ADCC) mediated by natural killer (NK) cells is considered a major contributor to antitumor efficacy [19, 20, 21, 22, 23]. Current EGFR‐targeting IgG antibodies include nimotuzumab, cetuximab, and panitumumab. Among these, humanized IgG1 nimotuzumab shows strong ADCC activity, which is superior to the IgG2 of panitumumab [24], with lower immunogenicity than that of cetuximab [25].

NK cells, which are the first‐line effectors of the innate immune system, directly lyse malignancies via perforin/granzyme release and death receptor activation [26]. However, the immunosuppressive microenvironment of PDAC severely compromises endogenous NK cell function [27, 28]. Adoptively transferred NK cells have a favorable safety profile in solid tumors [29, 30]; however, their efficacy in PDAC is limited by poor persistence and tumor infiltration [31, 32].

In this study, we proposed a synergistic strategy combining nimotuzumab with adoptively transferred NK cells to address the limited efficacy of therapies targeting KRAS‐driven cancers and boost NK cell antitumor activity in PDAC. This approach, which activates ADCC mediated by CD16 in NK cells and EGFR overexpression in PDAC cells, offers a promising therapeutic strategy for PDAC treatment.

## 2 Results

### 2.1 KRAS Mutations, EGFR Overexpression, and Low NK Cell Infiltration Correlate With Poor Prognosis in PDAC

**FIGURE 1:**
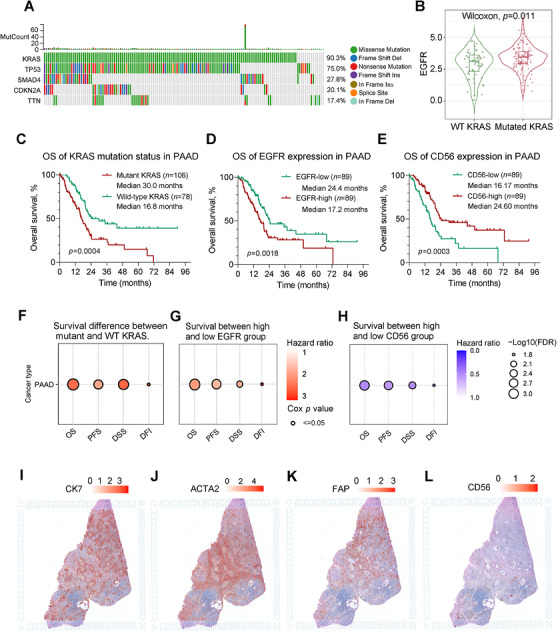
Evaluation of EGFR as an ADCC target in KRAS‐mutant PDAC. (A) Top five most frequently mutated genes in TCGA–PAAD. (B) Association between KRAS mutation status and EGFR expression in TCGA–PAAD. (C) Correlations of KRAS mutations, (D) EGFR expression, and (E) NK cell infiltration with overall survival in TCGA–PAAD. The survival statistical significance was analyzed via log‐rank test. (F) Associations of KRAS mutation status, (G) EGFR expression levels, and (H) NK cell infiltration density with patient survival outcomes (OS/PFS/DSS/DFI). (I–L) Spatial transcriptomic analysis from CROST database depicting: (I) expression of the PDAC tumor marker CK7, (J and K) fibroblast markers (ACTA2 and FAP), and (L) distribution of CD56^+^ NK cells within PDAC microenvironments. ADCC, antibody‐dependent cellular cytotoxicity; DFI, disease‐free interval; DSS, disease‐specific survival; NK, natural killer; OS, overall survival; PDAC, pancreatic ductal adenocarcinoma; PFS, progression‐free survival.

Analysis of TCGA–PAAD data identified KRAS as the most frequently mutated gene (90.3%; Figure 1A), with mutant tumors exhibiting significantly elevated EGFR expression (*p* = 0.011; Figure 1B). Survival analyses demonstrated significantly worse outcomes in KRAS‐mutant patients (*p* = 0.0004; Figure 1C). High EGFR expression correlated with reduced median OS (*p* = 0.0018; Figure 1D). Conversely, robust NK cell infiltration (CD56^+^) predicted favorable prognosis (*p* = 0.0003; Figure 1E). To integrate these findings, we explored the biological interplay between these variables. Results showed that KRAS wild‐type tumors exhibited significantly higher infiltration of both CD16^+^ and total NK cells than KRAS‐mutant tumors (Figure S1A), whereas high EGFR expression exhibited a trend toward reduced CD16^+^ NK cell scores (Figure S1B). This indicates that KRAS‐mutant and EGFR‐high tumors often harbor an “immune‐cold” microenvironment with deficient NK cell recruitment. To clarify their combined prognostic impact, a multivariate Cox regression analysis was performed (Figure S1C). The results identified high EGFR expression (HR = 1.70, *p* = 0.017) and age (HR = 1.34, *p* = 0.013) as independent risk factors for poor prognosis, whereas NCAM1 (CD56) expression remained a significant independent protective factor (HR = 0.56, *p* = 0.015). These trends were consistent across progression‐free survival, disease‐specific survival, and disease‐free interval endpoints (Figure 1F–H).

CROST is a comprehensive spatial transcriptomic database containing 1033 datasets across species and diseases. An analysis of PDAC spatial transcriptomic data from CROST first revealed an extensive distribution of tumor cells, as marked by CK7 expression (Figure 1I). Within this tumor ecosystem, we identified a widespread presence of fibrotic marker‐positive cells, including ACTA2^+^ myofibroblasts (Figure 1J) and FAP^+^ fibroblasts (Figure 1K). By contrast, CD56^+^ NK cells were restricted to the peripheral regions (Figure 1L). This spatial pattern demonstrates that pervasive stromal fibrosis associated with tumor cells forms a physical barrier that sequesters NK cells at the tumor margins, functionally impairing their cytotoxic infiltration into the tumor interior.

### 2.2 Nimotuzumab Potentiates NK Cells to Specifically Target EGFR‐Positive Tumor Cells

Flow cytometry confirmed a high‐purity population of CD3^−^/CD56^+^ cells (Figure S2A), with the CD56dimCD16^+^ subset comprising over 90% of the total (Figure S2B). The functional competence of these effectors was validated by a high expression of the activating receptors NKG2D (Figure S2C) and NKp46 (Figure S2D). The cytotoxic competence of these expanded NK cells was assessed using CD107a degranulation assays against AsPC‐1 cells (Figure S2E). Based on dose–response profiling across multiple effector‐to‐target (E:T) ratios, an optimal ratio of 10:1 was selected for all subsequent experiments (Figure S2F).

**FIGURE 2:**
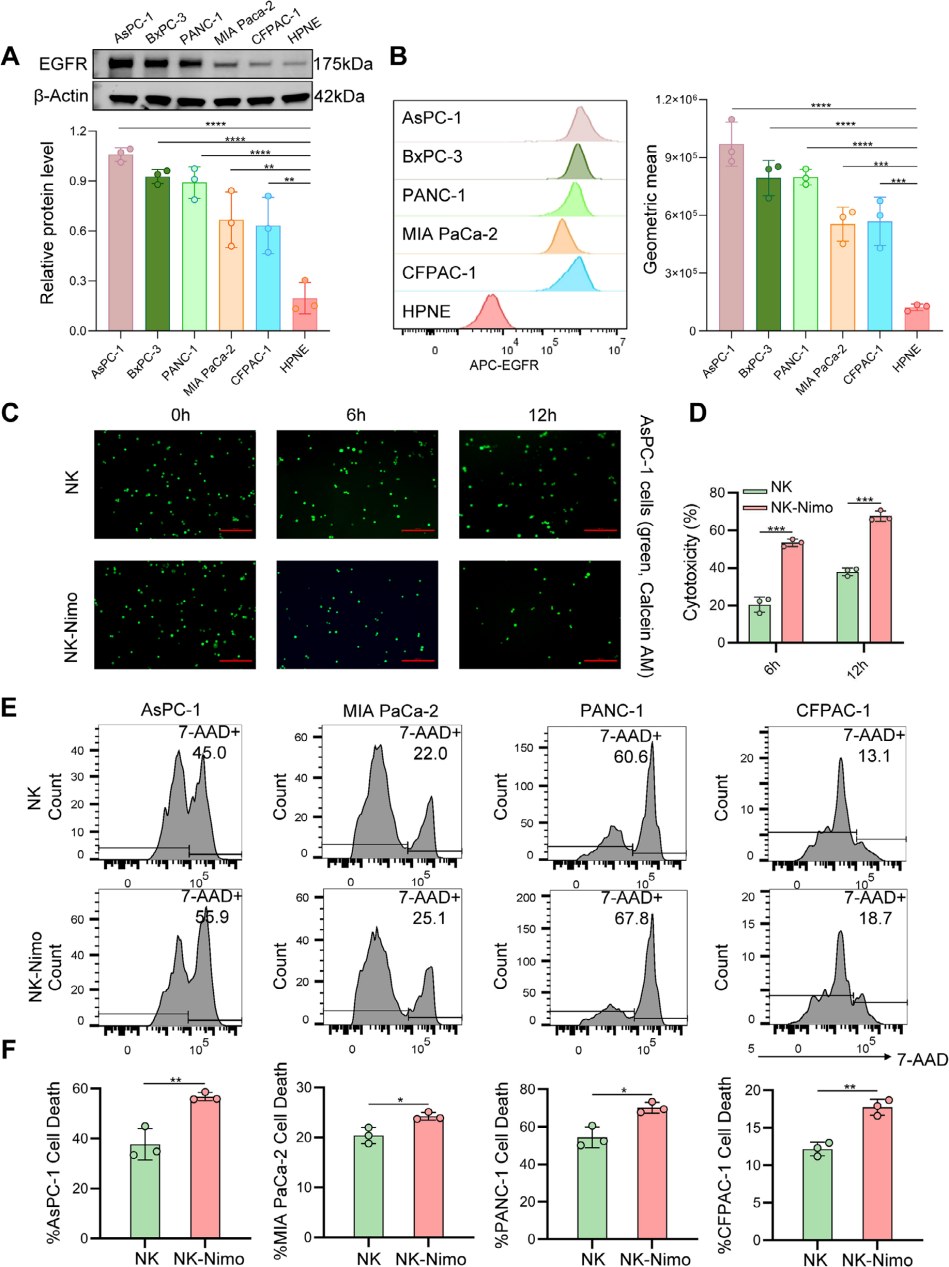
In vitro exploration of nimotuzumab combined with NK cell‐mediated cytotoxicity in pancreatic cancer. (A) EGFR protein expression in normal human pancreatic epithelial cells (HPNE) and five PDAC cell lines by Western blot. (B) Flow cytometry analysis of surface EGFR expression. (C) Time‐dependent cytotoxicity assay. Calcein‐AM‐labeled AsPC‐1 cells were co‐cultured with NK cells at 10:1 E:T ratio ± nimotuzumab, with viability measured at 0, 6, and 12 h. Scale bar = 200 µm. (D) Quantitative comparison of cytotoxicity between combination therapy and NK‐only groups. (E) Target cell death by CFSE/7‐AAD staining after 4 h co‐culture (7‐AAD^+^ in CFSE^+^ population), with (F) quantitative assessment. Data are presented as mean ± SD. The three individual data points in each group represent independent biological replicates (*n* = 3). The statistical significance was analyzed via one‐way ANOVA and *t*‐test. ^*^ *p* < 0.05, ^**^ *p* < 0.01, ^***^ *p* < 0.001, ^****^ *p* < 0.0001. E:T, effector‐to‐target; Nimo, nimotuzumab; NK, natural killer; PDAC, pancreatic ductal adenocarcinoma.

To evaluate the potential of EGFR as a target molecule for ADCC in PDAC, we assessed its expression in five PDAC cell lines and the normal pancreatic ductal epithelial cell line hTERT‐HPNE. EGFR expression was evaluated using Western blotting (Figure 2A) and flow cytometry (Figure 2B). Geometric mean normalization revealed differential EGFR expression across the tested cell lines, consistent with Western blotting and mRNA expression patterns from the Human Protein Atlas PAAD dataset (Figure S3A). All five PDAC cell lines exhibited higher EGFR expression than hTERT‐HPNE cells, with AsPC‐1 exhibiting the highest level and MIA PaCa‐2 and CFPAC‐1 exhibiting relatively low expression.

AsPC‐1 cells were labeled with calcein‐AM and co‐cultured with NK cells in the presence or absence of nimotuzumab. Compared with that in the NK cell‐only group, the number of AsPC‐1 cells in the combination group (NK‐Nimo group) was significantly reduced at 6 and 12 h (Figure 2C). Statistical quantification confirmed a significant increase in cytotoxicity (Figure 2D).

In addition to AsPC‐1 cells, nimotuzumab‐enhanced cytotoxicity was consistently observed in all the tested KRAS‐mutant PDAC cell lines. To evaluate the functional impact of nimotuzumab on ADCC, we quantified target cell killing using CFSE/7‐AAD co‐staining across four KRAS‐mutant PDAC cell lines (AsPC‐1, MIA PaCa‐2, PANC‐1, and CFPAC‐1) (Figure 2E). Our gating strategy (Figure S4A) identified dead target cells as the CFSE^+^7‐AAD^+^ population within the CFSE^+^ gate. Quantitative analysis showed that nimotuzumab significantly enhanced NK‐mediated cytotoxicity in all the tested KRAS‐mutant PDAC cell lines (Figure 2F). Notably, the magnitude of enhancement exhibited a strong positive correlation with baseline EGFR expression levels, being most prominent in high‐EGFR AsPC‐1 cells and minimal in low‐EGFR MIA PaCa‐2 cells (Figure S4B,C). This was supported by absolute quantification (Figure S5A), which exhibited a higher EGFR density in AsPC‐1 (∼2.4 × 10^7^ molecules/cell) than in MIA PaCa‐2 (∼5.8 × 10^6^ molecules/cell; Figure S5B,C). Although minor quantitative discrepancies were observed, this proportional relationship was broadly consistent and likely attributable to differences in baseline killing susceptibility among the cell lines.

To address whether the nimotuzumab‐mediated potentiation of cytotoxicity is a unique property or generalizable effect shared by other EGFR‐targeting IgG1 antibodies, we performed a head‐to‐head comparison with cetuximab in the EGFR‐high AsPC‐1 cell line. LDH release assays demonstrated that nimotuzumab was not inferior to cetuximab in driving NK cell‐mediated lysis (Figure S6A), indicating that the enhancement of tumor‐specific killing is a robust functional outcome of EGFR‐directed ADCC.

Similar results were observed in KRAS‐mutant/EGFR‐high A549 cells, supporting the broader applicability of this strategy (Figure S7A).

### 2.3 Nimotuzumab Drives ADCC to Enhance NK Cell Activation

**FIGURE 3:**
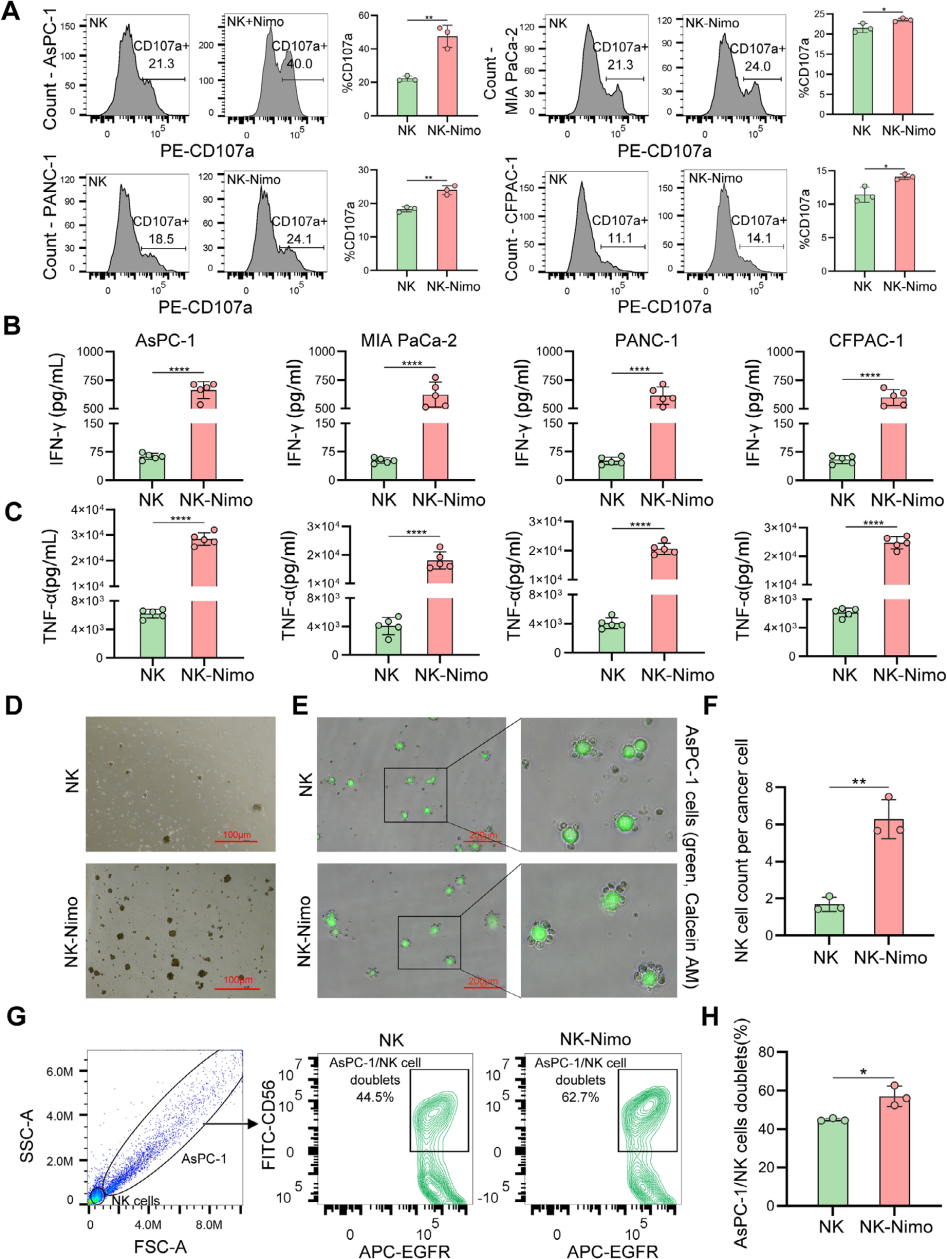
Nimotuzumab enhances NK cell activation via ADCC against PDAC. (A) NK cell degranulation measured by CD107a expression after 4 h co‐culture. IFN‐γ (B) and TNF‐α (C) secretion by activated NK cells after 24 h co‐culture. (D) NK cell aggregation in the absence or presence of nimotuzumab. Scale bar = 100 µm. (E) The effect of nimotuzumab treatment on NK cell recruitment to calcein‐AM‐labeled tumor cells was observed. Scale bar = 200 µm. (F) NK cell conjugation efficiency with tumor cells in the presence or absence of nimotuzumab treatment. (G) Flow cytometric analysis and (H) subsequent quantitative evaluation were performed to assess EGFR^+^ tumor cell‐CD56^+^ NK cell conjugate formation. Data are presented as mean ± SD. The three individual data points in each group represent independent biological replicates (*n* = 3). Groups compared using *t*‐test. ^*^ *p* < 0.05, ^**^ *p* < 0.01, ^****^ *p* < 0.0001; ns, not significant. ADCC, antibody‐dependent cellular cytotoxicity; IFN, interferon; Nimo, nimotuzumab; NK, natural killer; PDAC, pancreatic ductal adenocarcinoma; TNF, tumor necrosis factor.

To evaluate whether nimotuzumab activated NK cells, the NK activation marker CD107a was analyzed via flow cytometry after co‐culture with PDAC cells (Figure 3A). The results showed that the increased expression of CD107a induced by nimotuzumab was associated with a corresponding increase in the cytotoxicity of NK cells. In addition, although the basal levels of IFN‐γ and TNF‐α were similarly low in NK cells without nimotuzumab, the ELISA results showed that both cytokines were significantly increased upon nimotuzumab stimulation in the co‐culture systems of the four PDAC cell lines (Figure 3B,C). Further analysis of IFN‐γ secretion at different time points revealed that (Figure S8A) the combination of nimotuzumab and NK cells (NK‐Nimo) over a 24‐h period demonstrated significantly higher IFN‐γ production than the use of only NK. This advantage became more pronounced as the co‐culture duration extended, highlighting the synergistic effect of nimotuzumab in enhancing NK cell cytokine secretion. Furthermore, flow cytometric analysis of CD107a and IFN‐γ showed no significant differences between the nimotuzumab and cetuximab groups (Figure S8B,C), indicating that the induction of NK cell activation is a shared functional hallmark of EGFR‐directed ADCC.

To assess the interaction between NK and cancer cells in the presence or absence of nimotuzumab, we observed the co‐culture system under a microscope after 24 h. Notably, compared with those in the control group (NK cells alone), NK cells in the nimotuzumab‐stimulated group exhibited significantly increased clustering, indicating that nimotuzumab may enhance NK cell activation and aggregation (Figure 3D). To further investigate the targeting efficiency of NK cells, cancer cells were labeled with calcein‐AM and introduced into a co‐culture system. At the same E:T ratio, each cancer cell in the NK‐Nimo group attracted a greater number of NK cells than that in the NK cell‐only group (Figure 3E,F).

Next, we labeled NK cells with an anti‐CD56‐FITC antibody and AsPC‐1 cells with an anti‐EGFR‐APC antibody, followed by a 4‐h co‐culture period. For flow cytometric analysis, AsPC‐1 cells were gated based on forward and side scatter parameters (Figure 3G). The gated population exhibited complete APC positivity, confirming the success of the gating strategy. Within the AsPC‐1 cell population, cells that were positive for CD56‐FITC were identified as NK‐target cell conjugates. Results show a significant increase in the proportion of NK‐target cell conjugates in the NK‐Nimo group compared with the NK cell‐only group (Figure 3H). These findings indicate that nimotuzumab enhances the ability of NK cells to target and bind to cancer cells and significantly promotes NK cell activation, thereby potentially improving their cytotoxic efficacy.

To determine whether nimotuzumab‐mediated NK cell activation was dependent on CD16 engagement, primary NK cells were pretreated with a blocking antibody against CD16 (clone 3G8) before co‐culture with PDAC cells. LDH cytotoxicity assays (Figure S9A) and flow cytometric analyses of CD107a degranulation (Figure S9B) and IFN‐γ production (Figure S9C) demonstrated that CD16 blockade markedly attenuated the enhancement of NK‐mediated cytotoxicity and activation induced by nimotuzumab. To provide complementary evidence, we used the NK‐92 cell line, which is characterized by the absence of functional CD16 expression. Flow cytometry confirmed a predominantly CD56^+^/CD16^−^ phenotype (Figure S9D, with < 5% staining for CD16). In degranulation assays, although NK‐92 cells exhibited a minimal level of spontaneous CD107a expression (2%–5%) when co‐cultured with PDAC cells, the addition of nimotuzumab failed to induce any further increase in CD107a levels (Figure S9E,F). These results, which contrast with the robust activation observed in CD16^+^ primary NK cells, provide definitive evidence that nimotuzumab‐induced NK cell activation is strictly dependent on CD16‐mediated signaling.

### 2.4 Nimotuzumab Enhances NK Cell Infiltration and Tumor‐Specific Cytotoxicity in Three‐Dimensional Pancreatic Tumor Models

**FIGURE 4:**
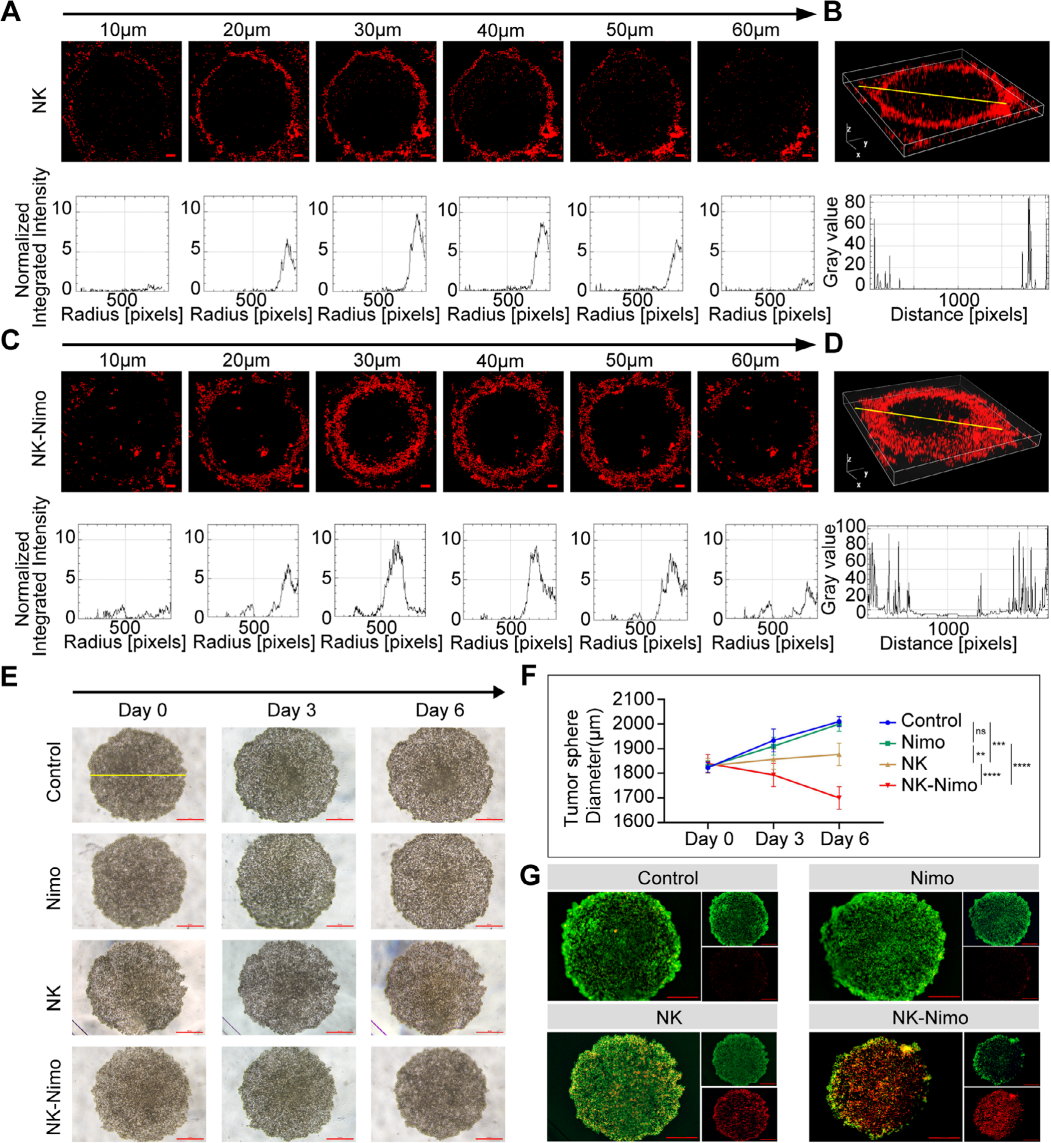
In vitro tumor penetration and antitumor efficacy toward AsPC‐1 tumor spheroids. Confocal Z‐stack imaging (10‐µm intervals) of Dil‐labeled NK cells co‐cultured with tumor spheroids for 12 h in the absence (A) or presence (C) of nimotuzumab, concentric fluorescence intensity analysis was conducted using ImageJ. Scale bar = 200 µm. (B and D) 3D reconstruction of Z‐stack images was performed. ImageJ‐based line‐scan analysis was used to quantify fluorescence intensity along spheroid diameters. (E) Microscopic images of AsPC‐1 spheroids that were treated with different formulations on Days 0, 3, and 6 under an inverted microscope with (F) quantitative analysis of spheroid diameter changes. Scale bar = 500 µm. (G) Viability assessment of spheroids treated with different formulations using calcein/PI live/dead staining. Scale bar = 500 µm. Data were presented as mean ± SD. Group differences were analyzed by one‐way ANOVA and Tukey's test. ^**^ *p* < 0.01, ^***^ *p* < 0.001, ^****^ *p* < 0.0001; ns, not significant. Nimo, nimotuzumab; NK, natural killer.

To investigate whether nimotuzumab‐mediated ADCC enhanced intratumoral NK cell penetration, a three‐dimensional tumor spheroid model of AsPC‐1 cells was established to mimic NK cell infiltration in solid tumors. After treatment with NK cells with or without nimotuzumab for 12 h, Z‐stack analysis using confocal laser scanning microscopy was performed to detect the intratumoral distribution of NK cells. As shown in Figure 4A,B, NK cells without nimotuzumab were confined to the periphery of tumor spheroids. By contrast, ADCC‐mediated targeting by nimotuzumab enabled NK cells to infiltrate deeper regions of the spheroids (Figure 4C,D). Further quantification using ImageJ revealed that the penetration depth in the nimotuzumab‐treated group reached approximately 500 µm. These results showed that EGFR‐directed ADCC activity critically enhanced NK cell infiltration into pancreatic tumor spheroids.

The cytotoxic efficacy against AsPC‐1 tumor spheroids was further evaluated by co‐incubation with the different treatment groups for 6 days. Tumor spheroid growth and structural changes were monitored every 3 days using optical microscopy, and the change in diameter was calculated pre‐ and post‐treatment (Figure 4E). The control and nimotuzumab‐only groups exhibited slight spheroid enlargement with no significant intergroup differences. Although NK cells alone inhibited spheroid growth, the combination of NK cells and nimotuzumab significantly induced spheroid shrinkage (Figure 4F). To validate cell viability, live/dead assays were performed and visualized by optical microscopy. Tumor spheroids in the control and nimotuzumab‐only groups exhibited intense green fluorescence (live cells) and negligible red fluorescence (dead cells), whereas those in the NK cell group exhibited limited peripheral red fluorescence. Importantly, the NK‐Nimo combination induced extensive red fluorescence (dead cells) throughout the spheroids (Figure 4G), indicating that ADCC enabled deep penetration and tumor eradication. These findings demonstrated that nimotuzumab synergizes with NK cells via EGFR‐directed ADCC to enhance intratumoral trafficking and retention, thereby achieving potent antitumor efficacy against PDAC spheroids.

To address the limitations of immunodeficient mouse models and validate the hypothesized immune cascade, we established an in vitro human immune cell crosstalk system. We observed that conditioned medium derived from nimotuzumab‐activated NK cells (via ADCC) was associated with increased expression of MHC class II (HLA‐DR) in THP‐1‐derived macrophage‐like cells (Figure S10A). Furthermore, this conditioned medium promotes macrophage recruitment (Figure S10B) and was accompanied by an increase in the proportion of CD69^+^ T cells within PBMC cultures (Figure S10C). Collectively, these observations provide preliminary evidence that NK cell activation may contribute to a broader immunostimulatory cascade involving macrophage activation and subsequent T‐cell responses (Figure S10D).

### 2.5 Biodistribution and Biosafety of NK Cells

**FIGURE 5:**
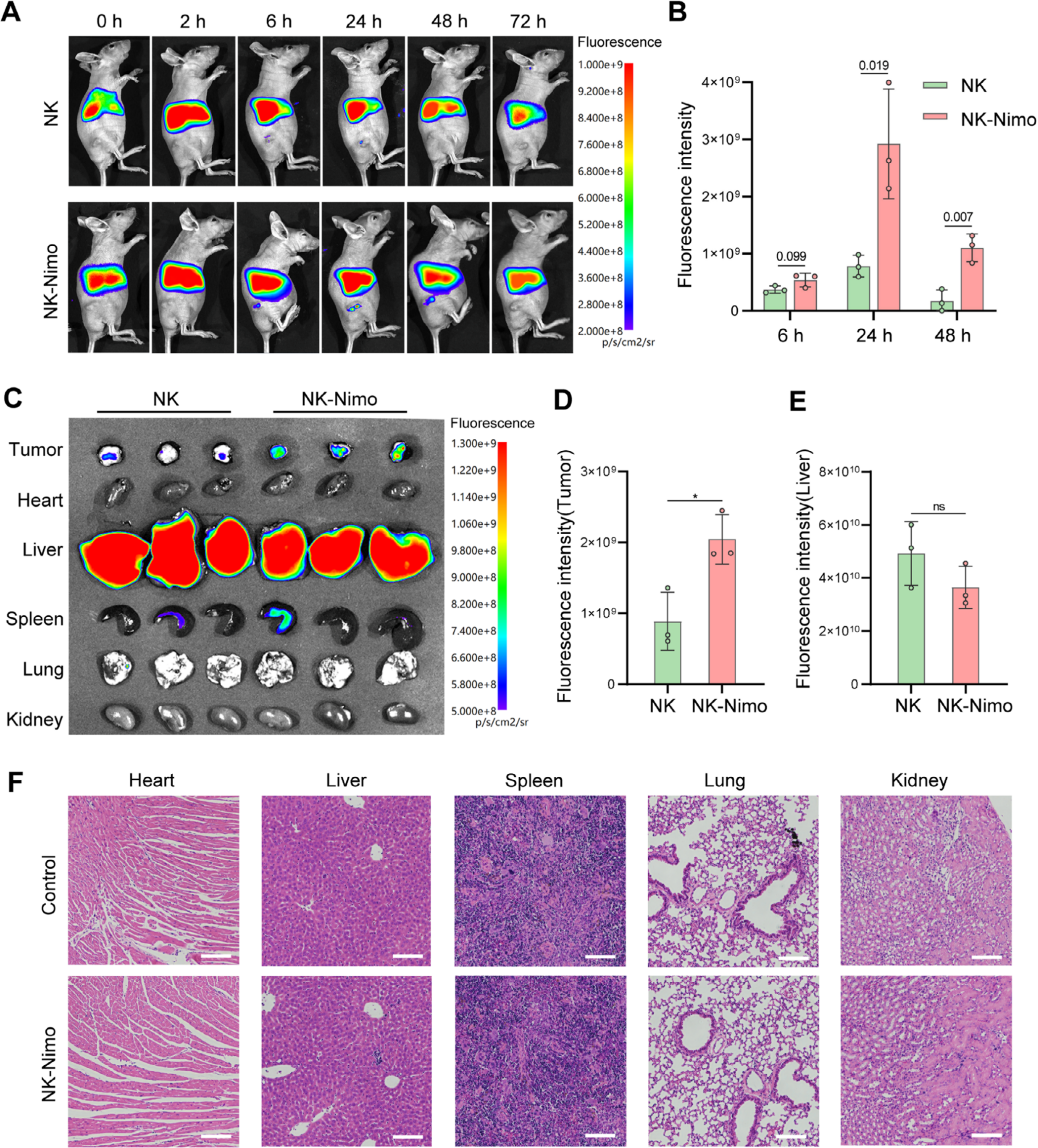
Biodistribution of NK cells and their targeting ability to PDAC in vivo. (A) Representative in vivo fluorescence images of BALB/c‐nu mice at various time points after intravenous injection of DIR‐labeled NK cells (NK‐only) and DIR‐labeled NK cells conjugated with nimotuzumab (NK‐Nimo). (B) Quantitative region‐of‐interest (ROI) analysis of tumor fluorescence intensity at 6, 24, and 48 h post‐injection, derived from the in vivo imaging data shown in panel A. (C) Ex vivo fluorescence images of dissected tumors and major organs obtained 24 h post‐injection from mice injected with DIR‐labeled NK cells. (D) Quantitative analysis of fluorescence intensity in ex vivo tumor tissues. (E) Quantitative analysis of fluorescence intensity in ex vivo liver tissues. (F) Representative hematoxylin and eosin (H&E)‐stained sections of major organs from mice treated with NK or NK‐Nimo. Scale bar = 50 µm. Data are presented as mean ± SD. Six mice were initially included in each group. Among them, three mice per group were used for in vivo fluorescence imaging and ROI quantification (A and B), whereas the remaining three mice per group were sacrificed for ex vivo imaging and tissue fluorescence analysis (C–E). Individual data points represent independent biological replicates. Statistical significance was determined by Student's *t*‐test. ^*^ *p* < 0.05; ns, not significant. Nimo, nimotuzumab; NK, natural killer; PDAC, pancreatic ductal adenocarcinoma.

To evaluate whether nimotuzumab enhances the tumor‐homing capacity of NK cells, mice received different injections. Tumor‐specific NK‐Nimo signals were detected as early as 6 h post‐injection and peaked at 24 h (Figure 5A,B). By contrast, tumor‐homing was barely detectable in the NK cell group at 6‐ and 24‐h time points. Fluorescence imaging and quantitative analysis of resected organs confirmed preferential accumulation of NK cells within tumor tissues in the NK‐Nimo group (Figure 5C,D). No statistically significant difference in liver fluorescence signals was observed between the two groups (Figure 5E), further highlighting the targeted nature of nimotuzumab‐mediated recruitment. In addition, hematoxylin and eosin (H&E) staining revealed no noticeable pathological changes in major organs (Figure 5F), and no skin alterations such as rashes were observed in any of the mice. These results show that NK‐Nimo did not cause significant toxicity in vivo.

### 2.6 Nimotuzumab and NK Cells Cooperatively Inhibit KRAS‐Mutant PDAC Tumor Growth in Mice

**FIGURE 6:**
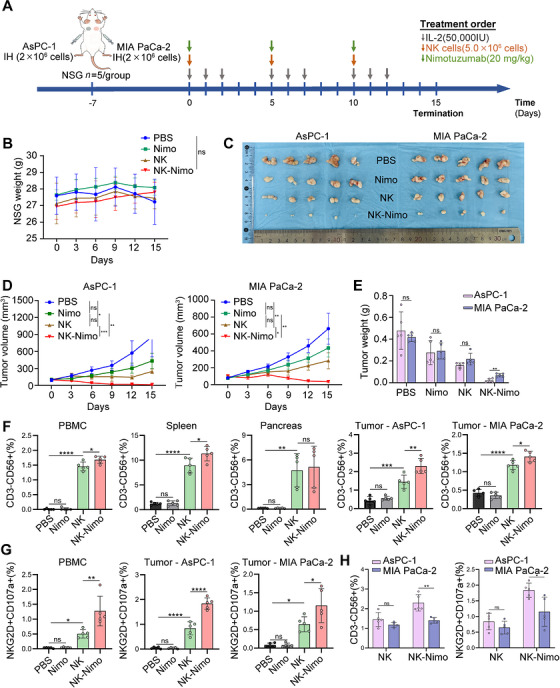
Antitumor efficacy of combination therapy in subcutaneous xenograft models. (A) Schematic of the experimental design: Heterogeneous subcutaneous xenografts were established by bilateral injection of AsPC‐1 cells (highest EGFR expression) and MIA PaCa‐2 cells (moderate EGFR expression) into NSG mice, followed by treatment with NK cells and/or nimotuzumab. (B) Body weight changes of mice during treatment. (C) Images of excised tumors after treatment completion. (D) Tumor volume dynamics of AsPC‐1 (left) and MIA PaCa‐2 (right) xenografts during treatment. (E) Comparative tumor weights at endpoint (*n* = 5/group). (F) Flow cytometry analysis of NK cell infiltration (human CD3^−^/CD56^+^ populations) in PBMCs, spleen, pancreas, and tumor tissues. (G) Flow cytometry quantification of activated NK cells (human NKG2D^+^/CD107a^+^) in PBMCs and bilateral tumors. (H) Comparative analysis of tumor‐infiltrating NK cell proportions and CD107a^+^ NK cell activation rates between bilateral xenografts. Data were presented as mean ± SD. Individual data points represent independent biological replicates (*n* = 5/group). Statistical significance was determined by one‐way ANOVA with Tukey's post hoc test for multigroup comparisons and a *t*‐test for pairwise comparisons. ^*^ *p* < 0.05, ^**^ *p* < 0.01, ^***^ *p* < 0.001, ^****^ *p* < 0.0001; ns, not significant. Nimo, nimotuzumab; NK, natural killer; PBMCs, peripheral blood mononuclear cells.

To evaluate the ADCC‐mediated antitumor activity of NK cells combined with nimotuzumab in vivo, we established a cell line‐derived xenograft tumor model in severely immunodeficient NSG mice. AsPC‐1 and MIA PaCa‐2 were subcutaneously injected into the bilateral flanks of the mice. One week later, the mice were divided into different treatment groups and administered NK cells and/or nimotuzumab for 5‐day cycles for a total of three treatment cycles (Figure 6A). Tumor growth and mouse body weight were monitored every 3 days to assess drug toxicity and changes in tumor size.

The results showed no significant differences in body weight between the treatment and untreated control groups, indicating that the combination therapy of NK cells and nimotuzumab did not cause significant toxicity (Figure 6B). After three treatment cycles, the mice were euthanized, and tumor sizes were measured. Although tumors in the nimotuzumab‐only group were smaller than those in the untreated group, the differences were modest and not statistically significant (Figure 6C,D). By contrast, compared with both the untreated and single‐treatment groups (NK‐only and nimotuzumab‐only), the NK‐Nimo group exhibited a significant reduction in bilateral tumor size.

Notably, although no significant differences in tumor size were observed between AsPC‐1 and MIA PaCa‐2 tumors in the control and single‐treatment groups, the NK‐Nimo group exhibited a less pronounced therapeutic effect on MIA PaCa‐2 tumors than on AsPC‐1 tumors (Figure 6E). This observation is consistent with our in vitro findings that ADCC efficacy is positively correlated with EGFR expression.

Next, we prepared single‐cell suspensions from the PBMCs, spleen, pancreas, and bilateral subcutaneous tumors of mice in each group. These suspensions were stained with human CD3 and CD56 to identify NK cells (Figure S11A) and with NKG2D and CD107a to detect activated NK cells (Figure S12A), followed by flow cytometric analysis. Results revealed significant NK cell infiltration in all examined tissues in the NK‐only and NK‐Nimo groups (Figure 6F). The highest proportion of NK cells was observed in the spleen, reaching approximately 10%, followed by the pancreas, where NK cells accounted for approximately 5% of the single‐cell suspension. Notably, the NK‐Nimo group exhibited a significantly higher proportion of NK cells in the spleen than the NK‐only group. A similar increasing trend was observed in the pancreas, although it did not reach statistical significance (*p* > 0.05), indicating that nimotuzumab may facilitate the accumulation of NK cells in these tissues.

In the PBMCs and AsPC‐1 subcutaneous tumors, the NK‐Nimo group showed a significantly higher proportion of activated NK cells than the other groups; however, this difference was not evident in MIA PaCa‐2 tumors (Figure 6G). To further compare the strength of the ADCC effects in bilateral subcutaneous tumors, we analyzed NK cell infiltration and activation. In the NK‐only group, NK cell infiltration and activation did not differ significantly between AsPC‐1 and MIA PaCa‐2 tumors. However, in the NK‐Nimo group, AsPC‐1 tumors with high EGFR expression exhibited significantly greater NK cell infiltration and activation than MIA PaCa‐2 tumors (Figure 6H). These findings highlight the importance of ADCC in suppressing tumor growth.

Furthermore, to evaluate the safety of repeated clinical‐mimetic dosing, major organs (heart, liver, spleen, lungs, and kidneys) were harvested at the end of the therapeutic study. Histopathological analysis via H&E staining revealed no evidence of structural damage, inflammatory infiltration, or cumulative toxicity in either the combination or control groups (Figure S13A), confirming the excellent systemic tolerability of long‐term nimotuzumab and NK cell co‐administration.

### 2.7 ADCC Targets Circulating Tumor Cells (CTCs) to Attenuate PDAC Lung Colonization

**FIGURE 7:**
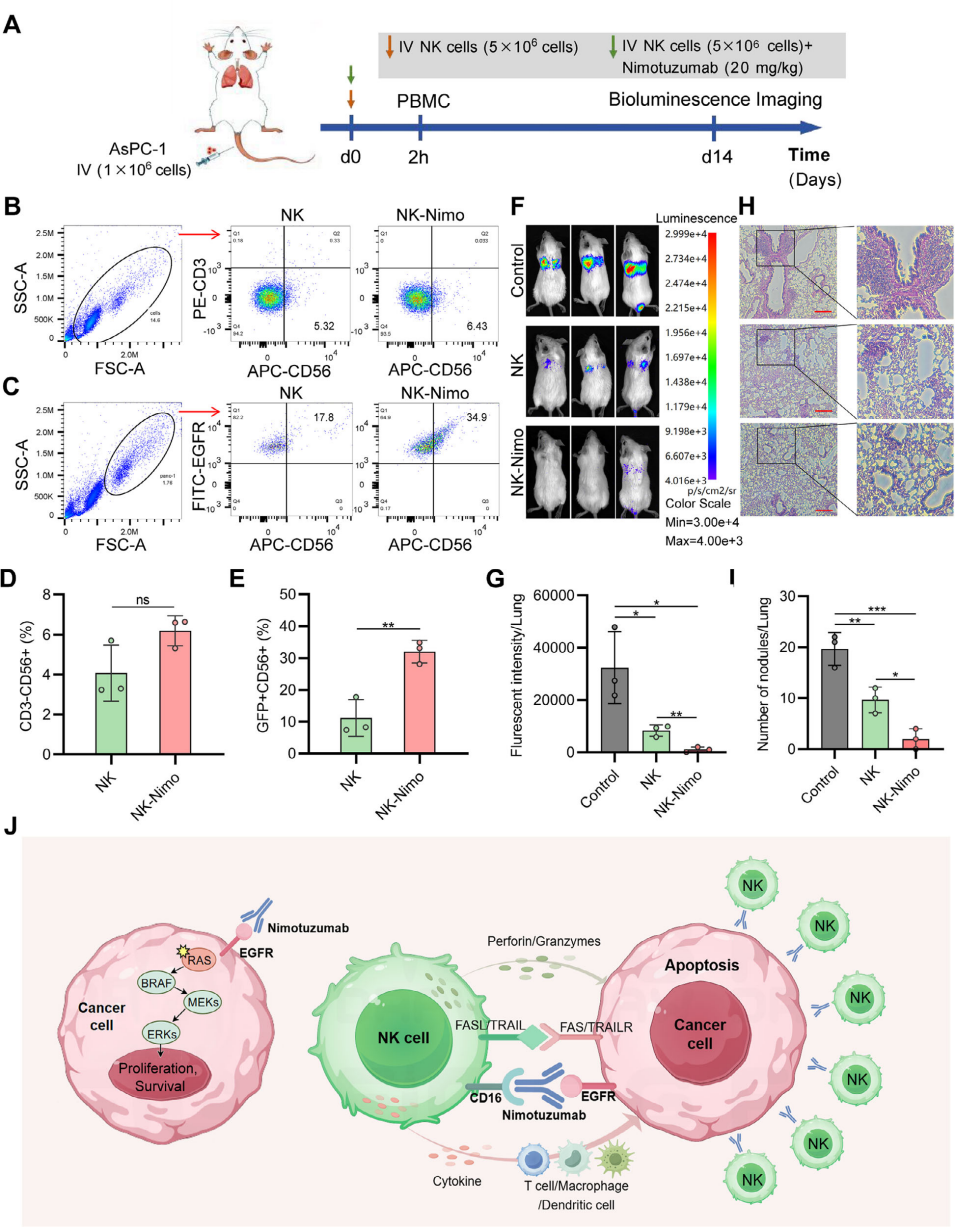
Combination therapy eliminates CTCs in peripheral blood. (A) Experimental design: NSG mice were intravenously inoculated with AsPC‐1‐LUC cells, followed by treatment with NK cells and/or nimotuzumab. (B and D) Peripheral blood analysis 2 h post‐injection: Flow cytometry quantification of NK cells (human CD3^−^/CD56^+^). (C and E) Proportion of target cells (EGFR^+^) bound by NK cells (EGFR^+^/CD56^+^). (F) Bioluminescence imaging and (G) quantification of lung metastases on Day 14. (H) H&E‐stained lung sections showing pathological lesions. Scale bar = 200 µm. (I) Quantification of metastatic lung nodules. (J) Proposed ADCC mechanism in KRAS‐mutant tumors. Despite persistent downstream pathway activation upon EGFR blockade, nimotuzumab recruits and activates NK cells for targeted tumor cell killing. Data were presented as mean ± SD. Six mice were initially included in each group. Among them, three mice per group were used for flow cytometric analyses of circulating NK cells and NK‐tumor cell conjugates (B–E), whereas the remaining three mice per group were used for bioluminescence imaging and histological evaluation of lung metastases (F–I). Individual data points represent independent biological replicates (*n* = 3 mice per group). Statistical significance was determined by a *t*‐test for pairwise comparisons and one‐way ANOVA with Tukey's post hoc test for multigroup comparisons. ^*^ *p* < 0.05, ^**^ *p* < 0.01, ^***^ *p* < 0.001; ns, not significant. CTCs, circulating tumor cells; Nimo, nimotuzumab; NK, natural killer; PBMCs, peripheral blood mononuclear cells.

To evaluate the adjuvant therapeutic potential of NK‐Nimo combination in postoperative settings, we established a CTC‐mediated metastasis model via tail vein injection of luciferase (LUC)‐labeled human PDAC cells (AsPC‐1) into NSG mice. Two independent experimental cohorts were used (Figure 7A), with peripheral blood collected 2 h post‐injection from the first cohort for analysis. Flow cytometric quantification assessed the engraftment efficiency of NK cells (human CD3^−^/CD56^+^) within mouse PBMCs along with the formation rate of NK‐tumor cell conjugates, identified as EGFR^+^/CD56^+^ dual‐positive events. Notably, although comparable proportions of human NK cells were detected in the NK‐only and NK‐Nimo combination groups (Figure 7B,D), the combination therapy exhibited significantly enhanced cytotoxic targeting, as evidenced by a higher frequency of EGFR^+^/CD56^+^ conjugates (Figure 7C,E).

To further evaluate metastatic colonization, a second cohort was analyzed on Day 14 using bioluminescence imaging to visualize and quantify lung metastases (Figure 7F,G). Results showed that combination therapy significantly suppressed tumor metastasis. These findings were further validated via histopathological assessment using H&E staining and quantitative tumor nodule counting (Figure 7H,I).

Mechanistically, nimotuzumab serves as a molecular bridge—binding EGFR overexpressed in PDAC cells (irrespective of KRAS status) and engaging CD16 receptors on NK cells to activate ADCC (Figure 7J).

## 3 Discussion

PDAC remains a formidable therapeutic challenge because of its aggressive biology [33] and resistance to conventional therapies [3]. This study demonstrated that the combination of nimotuzumab, an EGFR‐targeting mAb, with adoptive NK cell therapy exhibited potent therapeutic efficacy against KRAS‐mutated PDAC via ADCC. Our findings revealed the following mechanisms through which nimotuzumab enhances NK cell function: (1) serving as a molecular bridge to facilitate NK‐tumor cell interactions; (2) improving tumor penetration by promoting NK cell infiltration into dense PDAC stroma; and (3) amplifying NK cell effector activity, leading to robust tumor cell lysis. These results provide critical insights into the therapeutic potential of ADCC‐based strategies for KRAS‐mutant PDAC patients, a population historically excluded from EGFR‐targeted therapies [34].

The near‐universal presence of KRAS mutations in PDAC limits EGFR‐targeted therapies because of constitutive activation of downstream signaling [35]. Unlike small‐molecule EGFR inhibitors (e.g., erlotinib [36, 37, 38]), which rely on intracellular signaling blockade, nimotuzumab serves as a bifunctional engager‐bridging NK cells to tumor cells via ADCC. This aligns with the clinical evidence that the therapeutic efficacy of cetuximab directly correlates with ADCC intensity, particularly in patients with high‐affinity FcγRIIIa variants [39]. Our experiments showed that nimotuzumab alone exhibited minimal cytotoxicity against KRAS‐mutated tumors, and its combination with NK cells significantly enhanced target cell killing. We investigated whether the observed combined effect was mediated by NK cell activation. The results revealed that nimotuzumab treatment promoted NK cell clustering and the formation of dense conjugates surrounding target cells, a phenomenon similar to that observed with trastuzumab in HER2^+^ breast cancer [40]. This was accompanied by an increased release of cytotoxic granules and elevated secretion of proinflammatory cytokines (IFN‐γ and TNF‐α), similar to the enhanced NK cell activation observed with rituximab in lymphoma [41]. Previous studies have demonstrated that the exposure of NK cells to nimotuzumab‐coated tumor cells upregulates CD137 expression, indicating polyfunctional NK cell activation [24].

To verify this mechanism, we used CD16‐negative NK‐92 cells as a control. The reduced response observed in NK‐92 cells in the combination group is primarily attributable to their lack of CD16‐mediated ADCC. Furthermore, blocking the CD16 receptor on primary NK cells with the 3G8 antibody completely abolished nimotuzumab‐mediated enhancement, with cytotoxicity falling back to the NK‐only baseline. These results confirm that CD16‐mediated ADCC is an indispensable driver of the observed synergy. Another factor that may influence ADCC efficacy is the polymorphism of FcγRIIIa (CD16a). The FcγRIIIa‐158V allele has been reported to exhibit higher affinity for IgG1 Fc fragments than the 158F variant [42], potentially leading to variability in antibody‐mediated immune responses. Future studies evaluating genotype‐specific ADCC responses may clarify the contribution of FcγRIIIa polymorphisms to nimotuzumab‐mediated NK cell activation.

Critically, this mechanism effectively bypasses KRAS‐driven drug resistance, as ADCC efficacy depends primarily on target antigen density and NK cell function, rather than on intracellular signaling pathways. The ADCC potency of nimotuzumab positively correlates with EGFR expression levels, aligning with its unique bivalent‐binding properties. As demonstrated by Garrido et al. [43], nimotuzumab requires an intermediate‐to‐high EGFR density for stable attachment to cell surfaces, whereas its binding becomes transient at low EGFR levels. This property underlies both its selective tumor targeting and EGFR density‐dependent effector functions, including ADCC. Our data showed that the combination therapy achieved remarkable tumor regression in KRAS‐mutated AsPC‐1 xenografts with high EGFR expression. However, attenuated effects were observed in MIA PaCa‐2 tumors with low EGFR expression, consistent with the established correlation between EGFR density and ADCC potency [44, 45]. Furthermore, a comparison with cetuximab confirms that, although FcγRIIIa‐mediated NK cell activation is a shared mechanism among EGFR‐targeting IgG1 antibodies, nimotuzumab offers a more favorable efficacy–safety profile. Its binding characteristics enable preferential targeting of EGFR‐high tumor cells, thereby promoting localized immune activation while minimizing off‐tumor toxicity.

Our findings demonstrated that nimotuzumab‐mediated ADCC significantly enhanced NK cell penetration into pancreatic tumor spheroids, addressing the limited infiltration typically observed with NK cells alone. This observation is particularly notable, considering the well‐documented barriers to immune cell infiltration in PDAC. For instance, Watt et al. [46] demonstrated that the dense stromal microenvironment of pancreatic tumors actively excludes NK cells through physical and chemical signals (e.g., CXCL12‐mediated suppression [47]), highlighting the intrinsic resistance of this malignancy to innate immune surveillance. Similarly, Kloss et al. demonstrated that cetuximab (anti‐EGFR)‐mediated ADCC enhanced NK cell cytotoxicity and penetration into head and neck squamous cell carcinoma spheroids [48]. However, unlike these hematologic or epithelial cancers, PDAC spheroids pose additional barriers due to dense stromal components [49], rendering the observed 500‐µm penetration depth particularly notable. The profound cytotoxic effect, evidenced by widespread tumor cell death throughout the spheroid, further supports that ADCC facilitates NK cell access and sustains their functional persistence in the tumor interior [30, 50]. Notably, although the spheroid diameter exhibited only modest changes, this likely reflects the structural nature of multicellular spheroids, in which dead cells and cellular debris can remain within the architecture for some time, indicating that size measurements may underestimate the extent of internal cell death.

In addition to enhancing local penetration, our in vivo imaging results revealed that nimotuzumab significantly promotes the targeted homing of NK cells to the tumor site. Although the dense stroma and high interstitial fluid pressure of PDAC typically restrict the systemic recruitment of immune cells [5], the superior fluorescence signals observed in resected tumors demonstrate that nimotuzumab effectively facilitates tumor localization. Notably, the lack of a significant difference in liver accumulation between the groups further confirms that this recruitment is antigen‐specific, rather than a result of nonspecific systemic distribution.

Beyond mediating direct cytotoxicity, nimotuzumab‐primed NK cells secrete immunomodulatory factors like IFN‐γ and TNF‐α, which can polarize macrophages toward an M1‐like phenotype [51] and help bridge innate and adaptive immunity [52, 53]. Our expanded in vitro observations provide exploratory insights into this concept. We observed that the secretome from the nimotuzumab‐mediated ADCC system appeared to promote the recruitment and M1‐like polarization of THP‐1‐derived macrophages, as reflected by increased MHC‐II expression. Furthermore, this activated environment appears to support the activation of T cells within the PBMC population. These findings remain exploratory and provide only preliminary support for the proposed immune cascade and therefore warrant further investigation using more physiologically relevant models.

The success of this combination therapy in preclinical models holds significant clinical promise. First, it extends the applicability of nimotuzumab to KRAS‐mutant PDAC, which accounts for more than 90% of all PDAC cases. Second, the use of autologous or allogeneic NK cells, which lack MHC restriction and pose a lower risk of graft‐versus‐host disease, offers scalable “off‐the‐shelf” immunotherapy. Clinically, this strategy may be particularly relevant for patients with EGFR‐expressing, KRAS‐mutant PDAC, a population with limited targeted treatment options [54]. Our xenograft model demonstrated robust antitumor efficacy against established tumors, supporting the potential use of this strategy in combination with standard chemotherapy for advanced PDAC. In parallel, our murine metastasis model further demonstrates the potential of this strategy in adjuvant settings, in which eliminating residual CTCs could prevent postoperative recurrence. Importantly, the lack of significant toxicity in treated mice, as indicated by stable body weight and organ function, supports the safety profile of this combination. This is critical for translation to clinical trials, where patients with PDAC often exhibit compromised immune function because of advanced disease or prior therapy. Furthermore, our in vitro experiments using the A549 lung cancer cell line showed that this strategy may be broadly applicable to other solid tumors exhibiting high EGFR expression and KRAS mutations.

Although our findings are compelling, several limitations warrant further consideration. From a mechanistic perspective, the long‐term efficacy of ADCC‐mediated therapy in PDAC may be hindered by tumor‐intrinsic immune escape mechanisms. On one hand, the proteolytic shedding of stress ligands (MICA/B) by metalloproteases (e.g., ADAM10/17) can lead to chronic NKG2D internalization and subsequent NK cell deactivation [55]. On the other hand, the adaptive upregulation of inhibitory ligands such as HLA‐E can directly suppress NK cell function through the NKG2A receptor [27], suggesting potential resistance mechanisms that may limit durability.

In addition, the reliance on immunodeficient NSG mice limited the ability to model adaptive immune interactions. The long‐term durability of the response was also not evaluated, and whether immune memory develops against tumor‐associated antigens remains unclear. Moreover, owing to the staged endpoint design of the biodistribution and metastasis experiments, some analyses were performed with relatively small animal numbers per group. Although consistent trends were observed, larger cohorts are needed to strengthen statistical power. Collectively, based on these promising preclinical results, future studies and clinical trials are required to evaluate the safety and efficacy of this combination therapy in patients with advanced PDAC.

## 4 Conclusion

The combination of nimotuzumab and NK cells demonstrated potent antitumor activity against KRAS‐mutant PDAC by enhancing ADCC activity. Preclinical evidence has shown that nimotuzumab‐mediated ADCC significantly improves NK cell infiltration, activation, and cytotoxicity, particularly in EGFR‐high tumors, leading to reduced tumor growth and metastasis. These findings highlight a promising therapeutic strategy for diseases with poor prognoses and limited targeted options.

## 5 Materials and Methods

### 5.1 Bioinformatic Analysis

The RNA‐seq profiles and clinical information of 178 PDAD samples were downloaded from The Cancer Genome Atlas (TCGA) website (https://portal.gdc.cancer.gov/) on November 1, 2024. Mutations were analyzed based on TCGA whole‐exome sequencing data. The correlation between EGFR and KRAS, as well as survival analysis, was performed using cBioPortal (http://www.cbioportal.org/) or GSCA (https://guolab.wchscu.cn/GSCA/#/). Using the integrated portal for tumor‐immune system interaction (TISIDB, http://cis.hku.hk/TISIDB/), we explored the correlation between EGFR expression and stromal/immune cell infiltration in tumor tissues using the “estimate” package based on TCGA–PAAD data. We further analyzed the spatial transcriptomic (CROST) database (https://ngdc.cncb.ac.cn/crost) to examine the spatial transcriptome profiles of NK cells and fibrotic markers in PDAC tissues.

### 5.2 Cell Lines

Human normal pancreatic ductal cells (hTERT‐HPNE), human PDAC cell lines (AsPC‐1, BxPC‐3, MIA PaCa‐2, PANC‐1, and CFPAC‐1), and human non–small cell lung cancer cells (A549) were obtained from the American Type Culture Collection (ATCC, USA). NK‐92 cells were obtained from Procell Life Science & Technology Co. Ltd. (Wuhan, China). Cells were cultured in RPMI‐1640 or DMEM supplemented with 10% FBS and antibiotics, and NK‐92 cells were maintained in a specialized medium containing IL‐2 (200 U/mL). All cell lines were authenticated by short tandem repeat (STR) profiling and confirmed to be free of mycoplasma contamination before use.

### 5.3 NK Cell Isolation and Expansion

PBMCs were isolated from fresh blood using Ficoll density gradient centrifugation. For expansion, PBMCs were cultured in autologous plasma using the IL‐21 NK cell system (Zhongying Bio, China), following the manufacturer's instructions. From Days 7 to 14, cell density was maintained at 1–2 × 10^6^ cells/mL by adjusting the medium daily. Expanded NK cells were harvested on Day 14 of the logarithmic growth phase.

### 5.4 Western Blot

Protein lysates were extracted from 1 × 10^6^ cells using RIPA buffer (Sigma). After quantification via BCA assay, samples were prepared in loading buffer (Invitrogen) and denatured by heating at 95°C for 5 min. Proteins were transferred onto PVDF membranes and probed with primary antibodies against EGFR (Absin, #abs149686) and tubulin (Sigma‐Aldrich, #A6782), followed by incubation with a mouse secondary antibody (Sigma). Quantification was performed using ImageJ software after ECL detection.

### 5.5 LDH Release Assay

The cytotoxic activity of primary NK cells was determined using an LDH Cytotoxicity Assay Kit (Beyotime, China). Following the indicated treatments, culture supernatants were collected and incubated with LDH detection reagent for 30 min in the dark. Absorbance was measured at 490 nm using a microplate reader. Cytotoxicity was calculated according to the instructions of the manufacturer.

### 5.6 Flow Cytometry

Cell surface markers were analyzed using a CytoFLEX flow cytometer (Beckman Coulter). PDAC cells were characterized by anti‐EGFR staining, and NK cell purity and activation were assessed using antibodies against CD3, CD16, CD56, IFN‐γ, TNF‐α, CD107a, NKG2D, and NKp46. THP‐1‐derived macrophages and T cells were evaluated using MHC II and CD3/CD69 staining, respectively (BioLegend). For ADCC assays, CFSE‐labeled PDAC cells were co‐cultured with NK cells in the presence or absence of nimotuzumab (10 µg/mL), and cytotoxicity was evaluated by 7‐AAD staining. NK‐tumor cell conjugation assays were performed by labeling NK cells with anti‐CD56 and tumor cells with anti‐EGFR before co‐culture.

### 5.7 Targeted Mass Spectrometry

Membrane proteins were isolated from AsPC‐1 and MIA PaCa‐2 cells via subcellular fractionation, and the extracellular domain‐specific peptide, IPLENLQIIR, was targeted using LC‐PRM‐MS. Absolute peptide moles were calculated by comparison with an SIL internal standard and then converted to molecules using Avogadro's number. The total molecular count was normalized to the initial cell input and further adjusted for extraction recovery to yield the final chemically defined EGFR density (molecules per cell) for each cell line.

### 5.8 ELISA

Cytokines were quantified using sandwich ELISA. Supernatants were collected after 24 h of co‐incubation of the effector and target cells (E:T ratio, 10:1) in 96‐well plates. The concentrations of IFN‐γ and TNF‐α were measured using the High Sensitivity Human IFN‐γ ELISA Kit (Elabscience, #E‐HSEL‐H0007) and MF‐Human TNF‐α ELISA Kit (Reed Biotech, #P01375), respectively.

### 5.9 Cell Proliferation and Apoptosis Assays

AsPC‐1 cells were labeled with Calcein‐AM (AbMole, USA), and A‐549 cells were stained with Dil dye (Thermo Fisher Scientific, USA) and seeded into 12‐well plates. NK cells were added at a 10:1 E:T ratio ± nimotuzumab (10 µg/mL). Fluorescence microscopy was used to observe the co‐cultures at specific time points, and the number of surviving cancer cells was quantified using the ImageJ software.

### 5.10 Three‐Dimensional Tumor Spheroid Assay

AsPC‐1 spheroids were formed in low‐adhesion 96‐well plates for 72 h before co‐culture with DiI‐labeled NK cells ± 10 µg/mL nimotuzumab for 12 h. Spheroids were fixed with 4% PFA and analyzed via confocal Z‐stack imaging (Nikon AX; 10 µm intervals), NK cell infiltration depth was quantified using NIS‐Elements (Nikon) and ImageJ. In addition, spheroid size was monitored on Days 3 and 6, and cell viability was assessed using Calcein‐AM/PI staining and fluorescence imaging.

### 5.11 In Vivo NK Cell Tracking and Biodistribution Analysis

Twelve male BALB/c‐nu mice (male, 6‐ to 8‐week‐old) bearing subcutaneous AsPC‐1 tumors (60–80 mm^3^) were randomized into two groups (*n* = 6/group). All mice were purchased from Shanghai Model Organisms Center Inc. (Shanghai, China) and housed under specific pathogen‐free (SPF) conditions with a 12 h light/dark cycle and ad libitum access to food and water. Animals were acclimatized for at least 7 days prior to experimentation. Mice received tail vein injections of DiR‐labeled NK cells alone (5 × 10^6^ cells in 100 µL) or a combination of DiR‐labeled NK cells (5 × 10^6^ cells) ± nimotuzumab (20 mg/kg). For endpoint analysis, a two‐stage design was applied. At 24 h post‐injection, three mice per group were randomly selected and euthanized for ex vivo fluorescence imaging of major organs (heart, liver, spleen, lungs, and kidneys) as well as tumors. The remaining three mice per group were monitored longitudinally, and in vivo fluorescence signals were recorded up to 72 h post‐injection. Both in vivo and ex vivo fluorescence imaging were performed using the AniView Spectrum imaging system.

### 5.12 Tumor Xenograft Models

Six‐ to eight‐week‐old male M‐NSG mice (NOD.Cg‐*Prkdc* ^scid^ *Il2rg* ^em1Smoc^, Cat. NO. NM‐NSG‐001) were purchased from Shanghai Model Organisms Center Inc. (Shanghai, China). Animals were housed under the same SPF conditions and acclimatization protocol as described above. M‐NSG mice were subcutaneously injected with AsPC‐1 and MIA PaCa‐2 cells (2 × 10^6^ each). When tumors reached 60–80 mm^3^, mice (*n* = 5/group) were treated with PBS, nimotuzumab (20 mg/kg), NK cells (5 × 10^6^), or a combination of NK cells and nimotuzumab. Treatments were administered in three 5‐day cycles, and NK‐treated groups received hIL‐2 (50,000 units/mouse) daily for the first 3 days of each cycle. At study completion, tumors and organs were harvested for flow cytometric analysis of NK cell infiltration and activation.

To evaluate CTCs and metastasis, LUC‐labeled AsPC‐1 cells (1 × 10^6^) were intravenously injected into M‐NSG mice. Mice were randomly assigned to three groups (*n* = 6/group) and treated via tail vein injection with vehicle control (PBS), 5 × 10^6^ human NK cells, or a combination of NK cells (5 × 10^6^) and nimotuzumab (20 mg/kg). A two‐stage endpoint design was applied. At 2 h post‐treatment, three mice per group were randomly selected and euthanized for peripheral blood collection and flow cytometric analysis of NK‐tumor cell conjugates. The remaining three mice per group were monitored longitudinally, and metastatic progression was assessed on Day 14 by bioluminescence imaging (AniView), followed by euthanasia. Lung tissues were then harvested for H&E staining to quantify metastatic lesions.

All animal experiments were approved by the Experimental Animal Center of Qingdao University (Approval No. 20240904NSG3220241225134) and performed in accordance with institutional guidelines, the principles of the 3Rs (Replacement, Reduction, and Refinement), and the ARRIVE guidelines.

### 5.13 Quantification and Statistical Analysis

Data are presented as mean ± SD. Statistical analyses were performed using GraphPad Prism (version 8.0.2.263). Differences between groups were assessed using Student's *t*‐test or one‐way ANOVA with Tukey's post hoc test. Survival analysis was performed using the Kaplan–Meier method with log‐rank tests. A *p*‐value < 0.05 was considered statistically significant.

## Author Contributions

R.X., X.L., and P.L. contributed equally to this work. R.X. participated in writing – original draft, methodology, investigation, and conceptualization. X.L. performed investigation, formal analysis, and writing – review and editing. P.L. and K.L. contributed to writing – review and editing, project administration, and methodology. J.W. managed resources and methodology. X.S., Z.L., and M.R. conducted validation and investigation. C.Z. and R.G. performed validation and data curation. H.R. contributed to supervision, writing – review and editing, and funding acquisition. All authors have read and approved the final manuscript.

## Funding

This study was supported by funding from the National Natural Science Foundation of China (82125026, 82303933, and 82330081), the Horizontal Project of Qingdao University (Grant No. RH2300001355), the “Intergovernmental International Cooperation on Science and Technology Innovation” Key Special Project of China (2024YFE0104100), the “Medicine Plus” Joint Research Program of Qingdao University (YX2024201), and the Taishan Scholars Foundation (tsqn202507391).

## Ethics Statement

The collection of human peripheral blood samples was carried out in accordance with ethical approval from the Medical Ethics Committee of the Affiliated Hospital of Qingdao University (QYFYEC2024‐18). Approval for all animal experiments was granted by the Experimental Animal Center of Qingdao University (20240904NSG3220241225134).

## Conflicts of Interest

The authors declare no conflicts of interest.

## Supporting information




**Supporting File 1**: mco270860‐sup‐0001‐SuppMat.docx

## Data Availability

RNA‐seq expression profiles and clinical data for TCGA–PAAD were obtained from The Cancer Genome Atlas (TCGA) via the Genomic Data Commons (GDC) portal (https://portal.gdc.cancer.gov/) on November 1, 2024. Somatic mutation data were derived from TCGA whole‐exome sequencing datasets. All datasets used in this study are publicly available, de‐identified, and were accessed in accordance with TCGA/GDC data‐sharing policies. Additional analyses were performed using cBioPortal (http://www.cbioportal.org/), GSCA (https://guolab.wchscu.cn/GSCA/#/), TISIDB (http://cis.hku.hk/TISIDB/), and CROST (https://ngdc.cncb.ac.cn/crost).
