# Supplementary material for: EGFR‐Targeting IgG1 Antibody Enhances NK Cell‐Mediated Tumor Killing in KRAS‐Mutant Pancreatic Cancer
Source: MedComm (2020). 2026 Jun 23;7(7):e70860. doi: 10.1002/mco2.70860 (PMC13291551; doi:10.1002/mco2.70860)

**Supplementary Information**

**EGFR-Targeting IgG1 Antibody Enhances NK Cell-Mediated Tumor Killing in KRAS-Mutant Pancreatic Cancer**

Ruoxi Xiao^1, 3^, Xiaoxiao Li^2^, Ping Li^4^, Junjin Wang^3, 5^, Xiaoyuan Sun^2^, Chenyang Zhao^1, 5^, Zimin Liu^2^, Ruining Gong^1, 2^, Minghan Ren^3^, Ke Lei^1^*, He Ren^1, 2, 5^*

1. Shandong Provincial Key Laboratory of Clinical Research for Pancreatic Diseases, Tumor Immunology and Cytotherapy, Medical Research Center, The Affiliated Hospital of Qingdao University, Qingdao, 266000, China.
2. Gastrointestinal Cancer Institute/Pancreatic Disease Institute, The Affiliated Hospital of Qingdao University, Qingdao, 266000, China.
3. Department of Clinical Medicine, Qingdao University, Qingdao, 266000, China.
4. Cheeloo College of Medicine, Qilu Hospital (Qingdao), Shandong University, Qingdao 266000, China.
5. Tumor Immunology and Cytotherapy of Medical Research Center, the Affiliated Hospital of Qingdao University, Qingdao 266000, China.

**Short running title:** EGFR-Targeted NK Cell ADCC in KRAS-Mutant PDAC

**Correspondence to**

Professor He Ren; herenrh@163. com

Professor Ke Lei; [leike@qdu.edu.cn](mailto:leike@qdu.edu.cn)

**Supplementary figure 1. Interaction Between EGFR Expression, KRAS Mutation Status, and NK Cell Infiltration in Pancreatic Cancer.** (A) Infiltration of Total and CD16+ NK Cells Across Different KRAS Mutation Status. (B) Correlation Between EGFR Expression and CD16+ NK Cell Infiltration. (C) Multivariate Cox Regression Analysis of Prognostic Factors in PDAC.

**
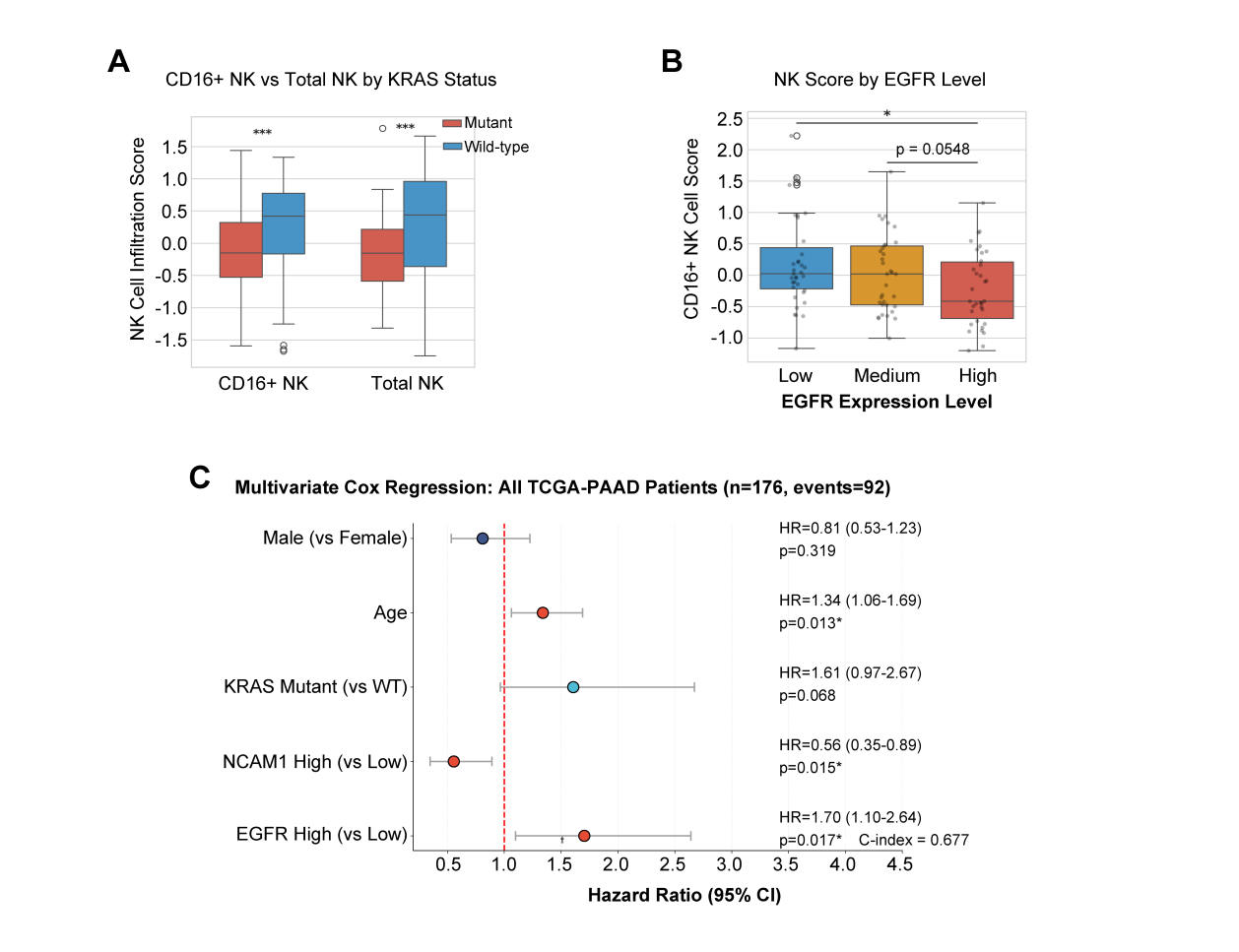
**

**Supplementary figure 2. Purity and cytotoxic activity assessment of NK cells.** (A) Flow cytometry analysis of NK cell purity (human CD3⁻/CD56⁺). (B-D) Representative flow cytometry histograms illustrating the surface expression of key activating receptors, including CD16 (B), NKG2D (C), and NKp46 (D) on the gated CD3-/CD56+ NK cell population. (E) NK cell activation (CD56⁺/CD107a⁺) and dose-dependent cytotoxicity at different E:T ratios after coculture with pancreatic cancer cells. (F) Statistical analysis of CD107a expression. NK, natural killer; E:T, effector-to-target.

**
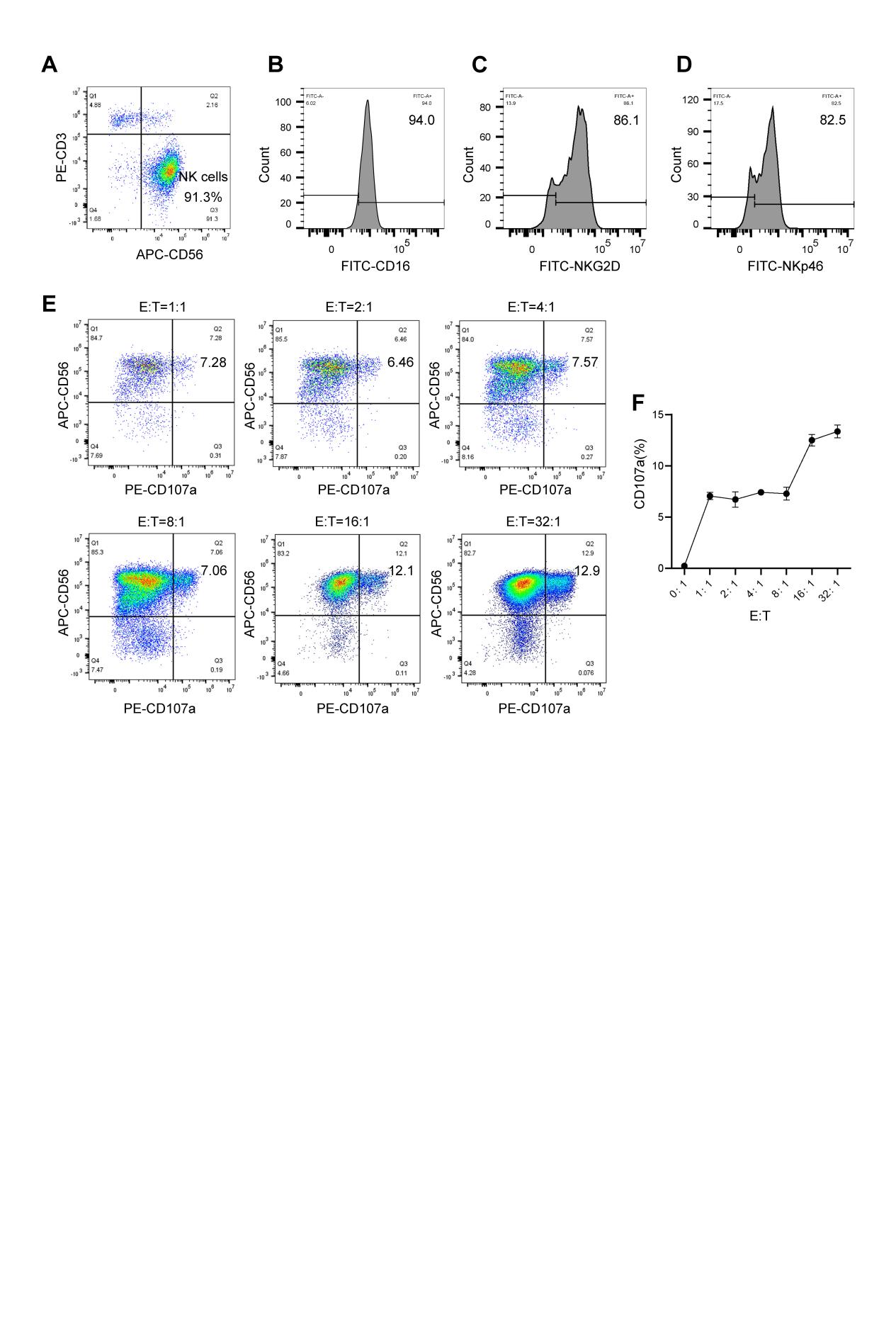
**

**Supplementary figure 3. RNA expression profiles of EGFR in human pancreatic cancer cell lines.** (A) RNA expression levels of EGFR in PDAC cell lines, sourced from the Human Protein Atlas database. PDAC, pancreatic ductal adenocarcinoma.


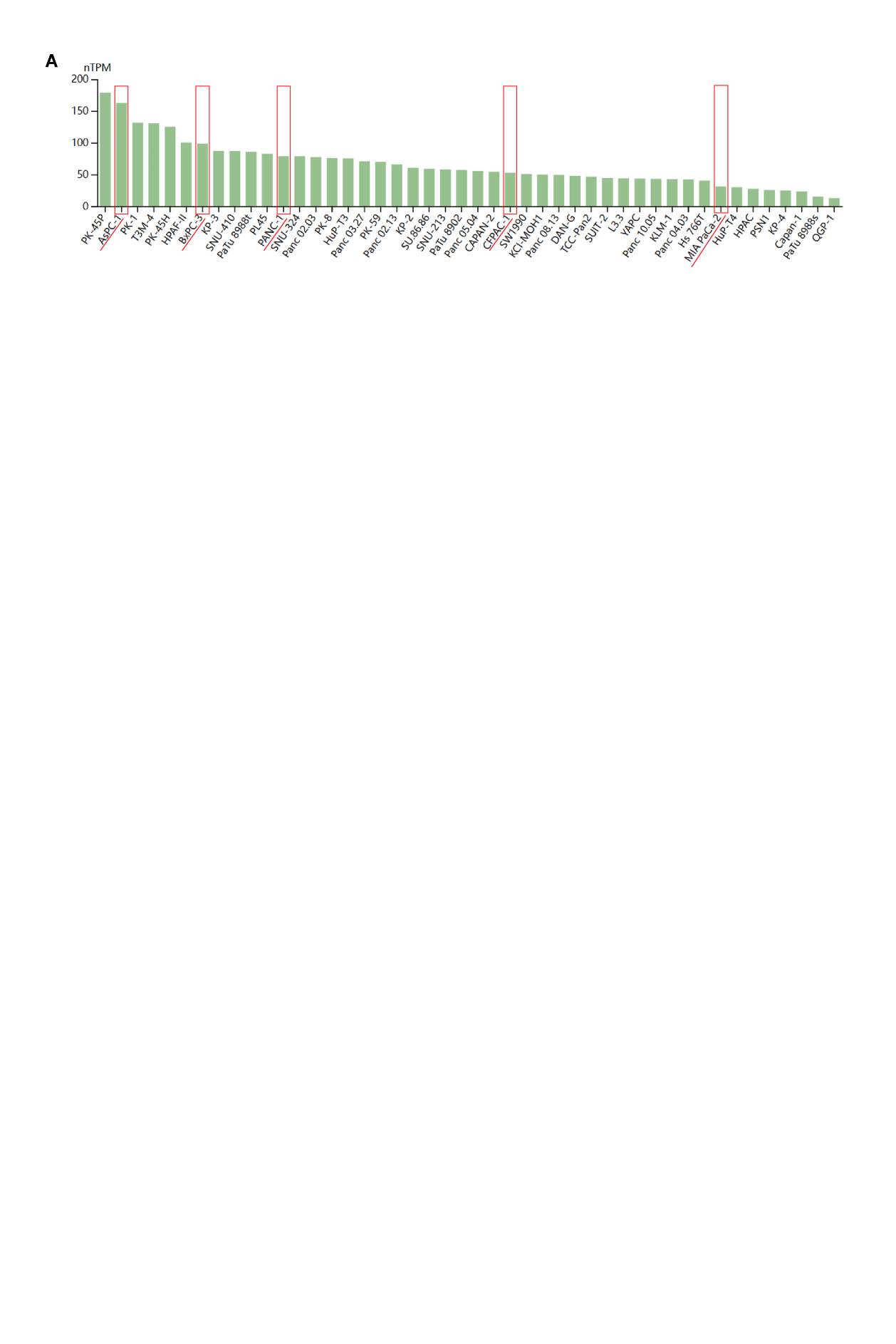


**Supplementary figure 4. Flow cytometric gating strategy and ADCC activity assessment.** (A) Gating strategy for CFSE/7-AAD flow cytometry to quantify ADCC activity. (B) Cytotoxicity assay of four PDAC-mutant cell lines quantified by 7-AAD staining. (C) ADCC-specific cytotoxicity was calculated as the difference in % cytotoxicity between NK-Nimo and NK alone groups for each cell line. Data were presented as mean ± SD. The three individual data points in each group represent independent biological replicates (n=3). Statistical significance was determined by t-test for pairwise comparisons and one-way ANOVA with Tukey’s post-hoc test for multi-group comparisons. *p < 0.05, **p < 0.01; ns, not significant. ADCC, antibody-dependent cellular cytotoxicity; PDAC, pancreatic ductal adenocarcinoma; NK, natural killer; Nimo, nimotuzumab.


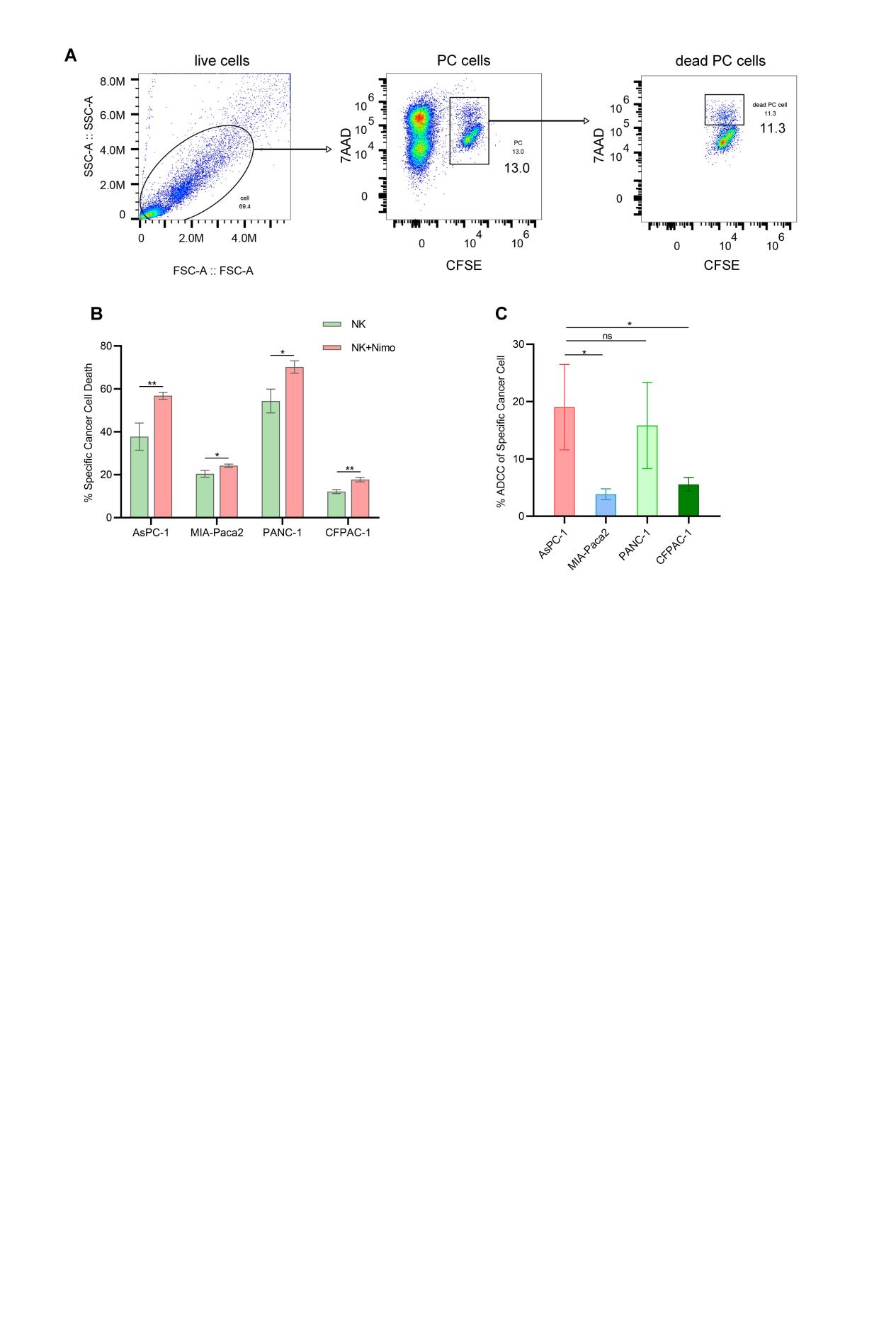


**Supplementary figure 5. Quantitation of EGFR Molecules in MIA Paca-2 and AsPC-1 Cell Lines Using a Targeted Mass Spectrometry Workflow.** (A) A schematic workflow of the targeted proteotypic peptide-based method for quantification. (B) The relative peak areas of the endogenous EGFR proteotypic peptide measured by SRM in AsPC-1 and MIA Paca-2 cells. (C) Normalized Quantification of EGFR Molecules per Cell. Using the internal standard standard curve, the peptide peak areas are converted to absolute molecule counts and normalized to the number of cells. AsPC-1 cells possess a much higher density of EGFR molecules per cell (approximately 2.4×10^7^) than MIA Paca-2 cells (approximately 5.8×10^6^).


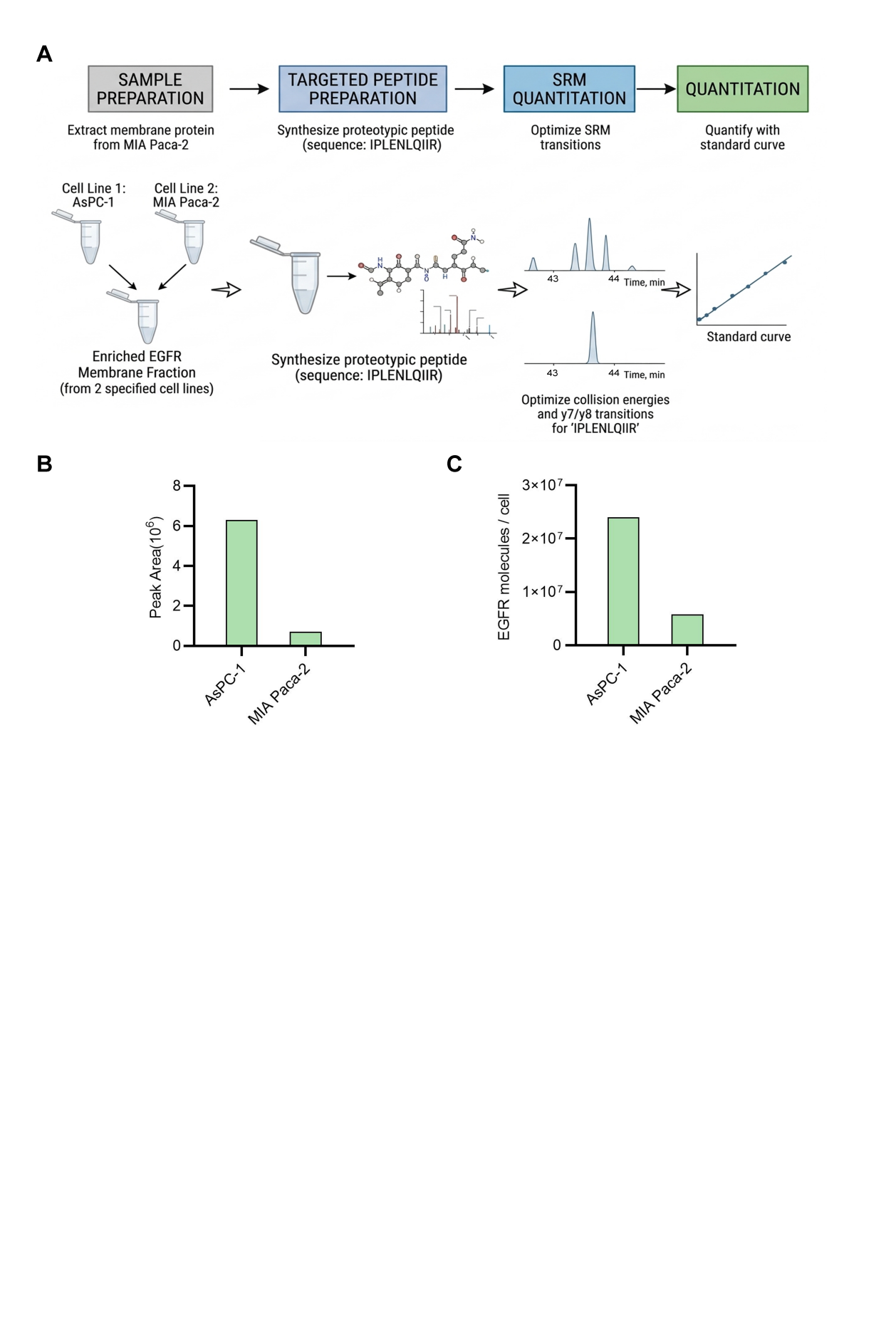


**Supplementary figure 6. Comparison of nimotuzumab- and cetuximab-mediated ADCC in PDAC cells.** Comparative analysis of NK cell-mediated cytotoxicity induced by nimotuzumab (10 μg/mL) and cetuximab (10 μg/mL) against AsPC-1 cells at indicated E:T ratios, as measured by LDH release assay. Data were presented as mean + SD. The three individual data points in each group represent independent biological replicates (n=3). Statistical significance was determined by t-test for pairwise comparisons and one-way ANOVA with Tukey’s post-hoc test for multi-group comparisons. *p < 0.05, ***p <0.001; ****p < 0.0001; ns, not significant. ADCC, antibody-dependent cellular cytotoxicity; PDAC, pancreatic ductal adenocarcinoma; NK, natural killer; Nimo, nimotuzumab; Cet, cetuximab.


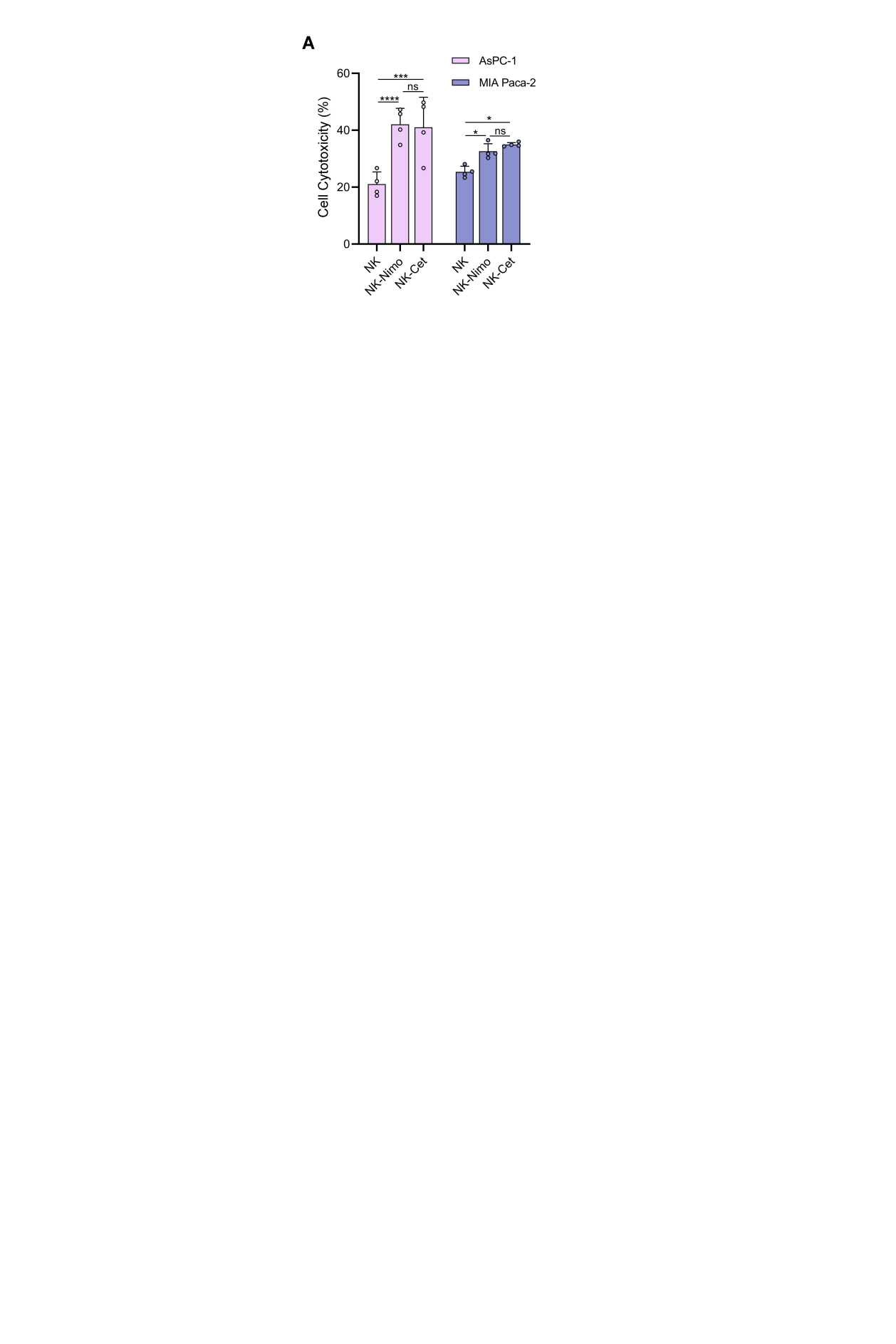


**Supplementary figure 7. ADCC efficacy against lung cancer cell line A549.** Time-dependent cytotoxicity assay of DiL-labeled A549 cells co-cultured with NK cells (E:T = 10:1) in the presence or absence of nimotuzumab, with quantitative analysis performed at 0, 12, and 24 h. Scale bar = 200 μm. Data were presented as mean + SD. Statistical significance was determined by one-way ANOVA with Tukey’s post-hoc test. ***p < 0.001, ****p < 0.0001; ns, not significant. NK, natural killer; Nimo, nimotuzumab.


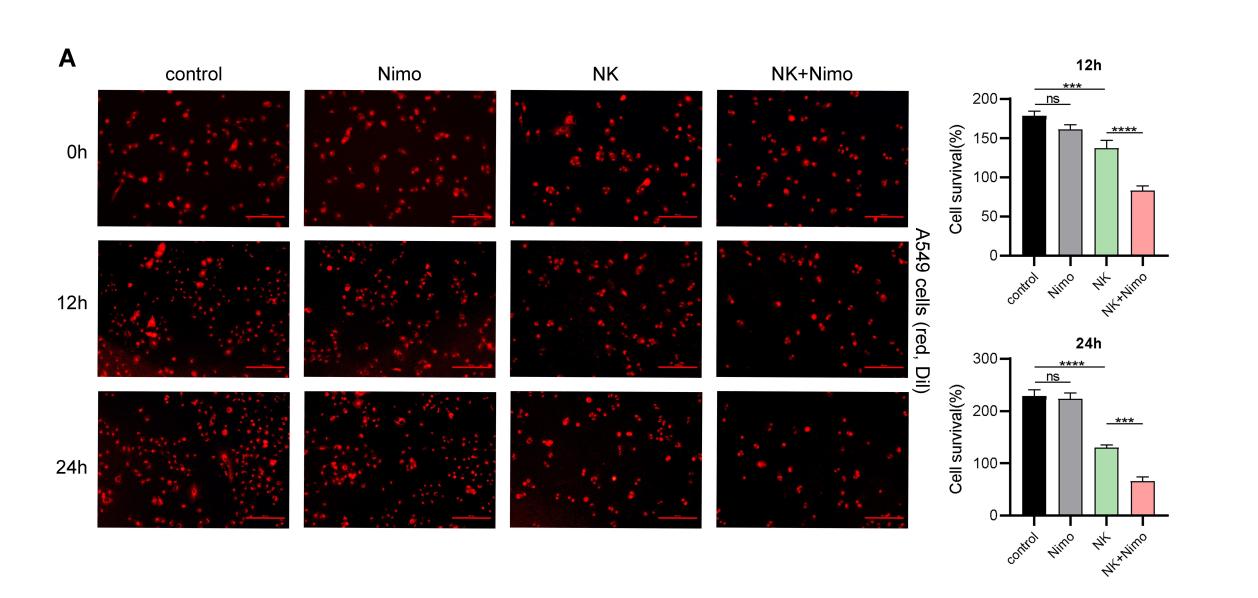


**Supplementary figure 8. Comparative analysis of NK cell activation markers and cytokine secretion induced by nimotuzumab and cetuximab. (A)** Time-dependent IFN-γ secretion by NK cells measured by ELISA. (B, C) Quantification of (B) CD107a degranulation and (C) intracellular IFN-γ production in NK cells after co-culture with AsPC-1 and MIA Paca-2 cells in the presence of nimotuzumab or cetuximab. Data were presented as mean + SD. The three individual data points in each group represent independent biological replicates (n=3). Statistical significance was determined by one-way ANOVA with Tukey’s post-hoc test. **p < 0.01, ****p < 0.0001; ns, not significant. NK, natural killer; Nimo, nimotuzumab.


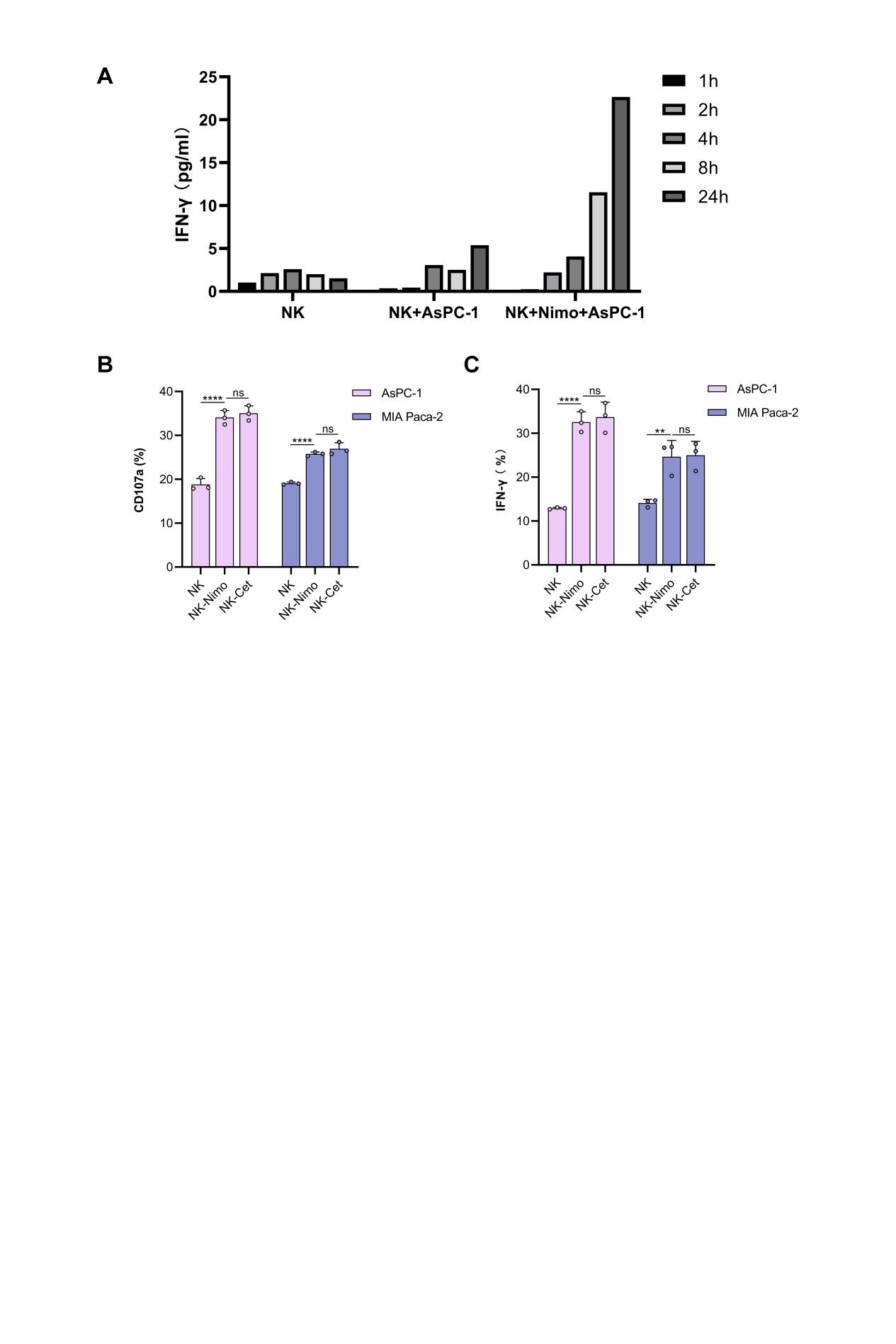


**Supplementary figure 9. Nimotuzumab-induced NK cell activation and cytotoxicity are largely dependent on CD16 signaling.** (A) Specific lysis of PDAC cells by primary NK cells with or without CD16 blocking antibody (3G8) pretreatment, as measured by LDH release assay. (B, C) Quantification of (B) CD107a degranulation and (C) intracellular IFN-γ production in primary NK cells following CD16 blockade. (D) Phenotypic characterization of NK-92 cells by flow cytometry confirming a CD56+/CD16- profile. (E, F) Representative flow cytometry plots (E) and statistical summary (F) of CD107a expression in NK-92 cells co-cultured with PDAC cells in the presence or absence of nimotuzumab. Data are presented as mean + SD from three independent experiments. The three individual data points in each group represent independent biological replicates (n=3). Statistical significance was determined by one-way ANOVA with Tukey’s post-hoc test for multi-group comparisons, and t-test for pairwise comparisons. *p < 0.05, **p < 0.01, ***p < 0.001; ****p < 0.0001; ns, not significant. NK, natural killer; Nimo, nimotuzumab; PDAC, pancreatic ductal adenocarcinoma.


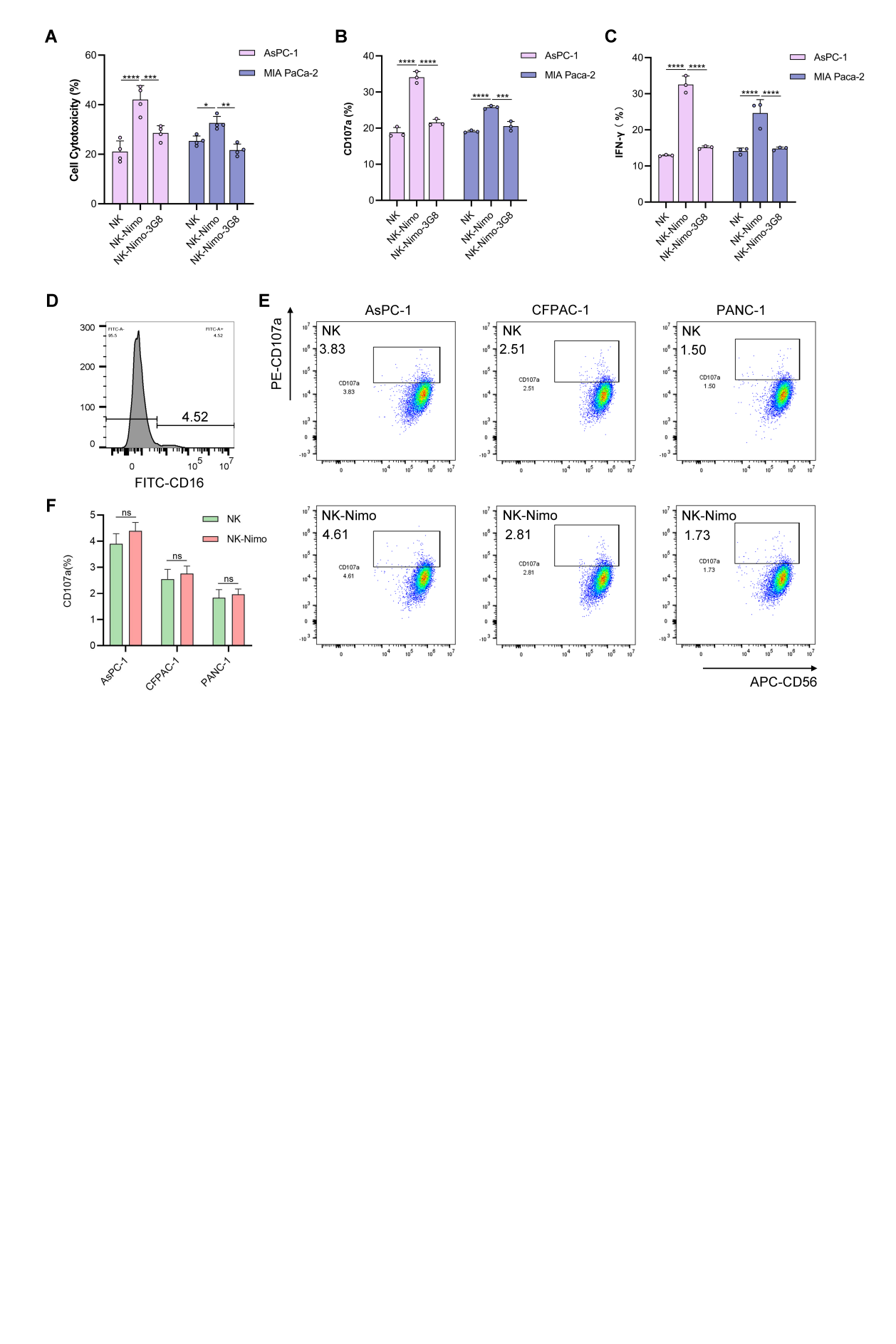


**Supplementary figure 10. Impact of Nimotuzumab-mediated ADCC on the modulation of the tumor immune microenvironment.** (A) Representative flow cytometry plots and statistical quantification of MHC II (HLA-DR) expression on PMA-pretreated THP-1 cells after 24 h incubation with supernatants from the indicated co-culture systems. (B) PMA-primed THP-1 cells (upper chamber) were exposed to conditioned media from different co-culture systems (lower chamber) in a transwell assay. Migrated cells were observed after 24 h. Scale bar = 100 μm. (C) Flow cytometric analysis and quantification of CD69 expression on CD3+ T cells following 24 h of co-culture with the indicated systems.(D) Proposed Model of the Immunostimulatory Cascade. Data were presented as mean + SD. Statistical significance was determined by one-way ANOVA with Tukey’s post-hoc test. *p < 0.05, **p < 0.01; ns, not significant. NK, natural killer; Nimo, nimotuzumab.

**
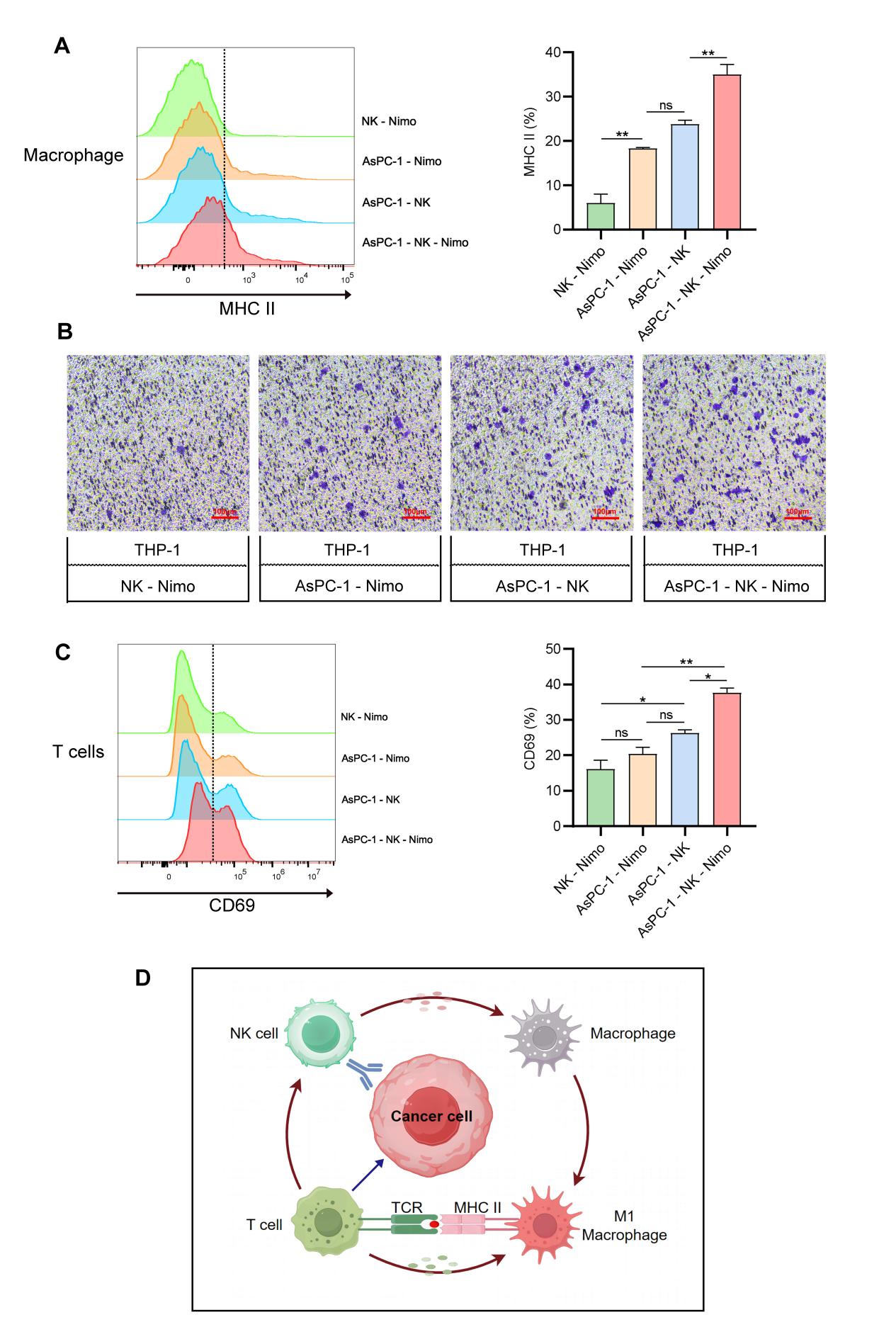
**

**Supplementary figure 11. NK cell infiltration across tissues in vivo.** Representative flow cytometry plots of human NK cells (human CD3⁻/CD56⁺) in (A) PBMCs, (B) spleen, (C) pancreas, (D) AsPC-1 and (E) MIA PaCa-2 tumor. Data were presented as mean + SD. Statistical significance was determined by one-way ANOVA with Tukey’s post-hoc test. *p < 0.05, **p < 0.01, ***p < 0.001, ****p < 0.0001; ns, not significant. NK, natural killer; Nimo, nimotuzumab; PBMCs, Peripheral blood mononuclear cells.


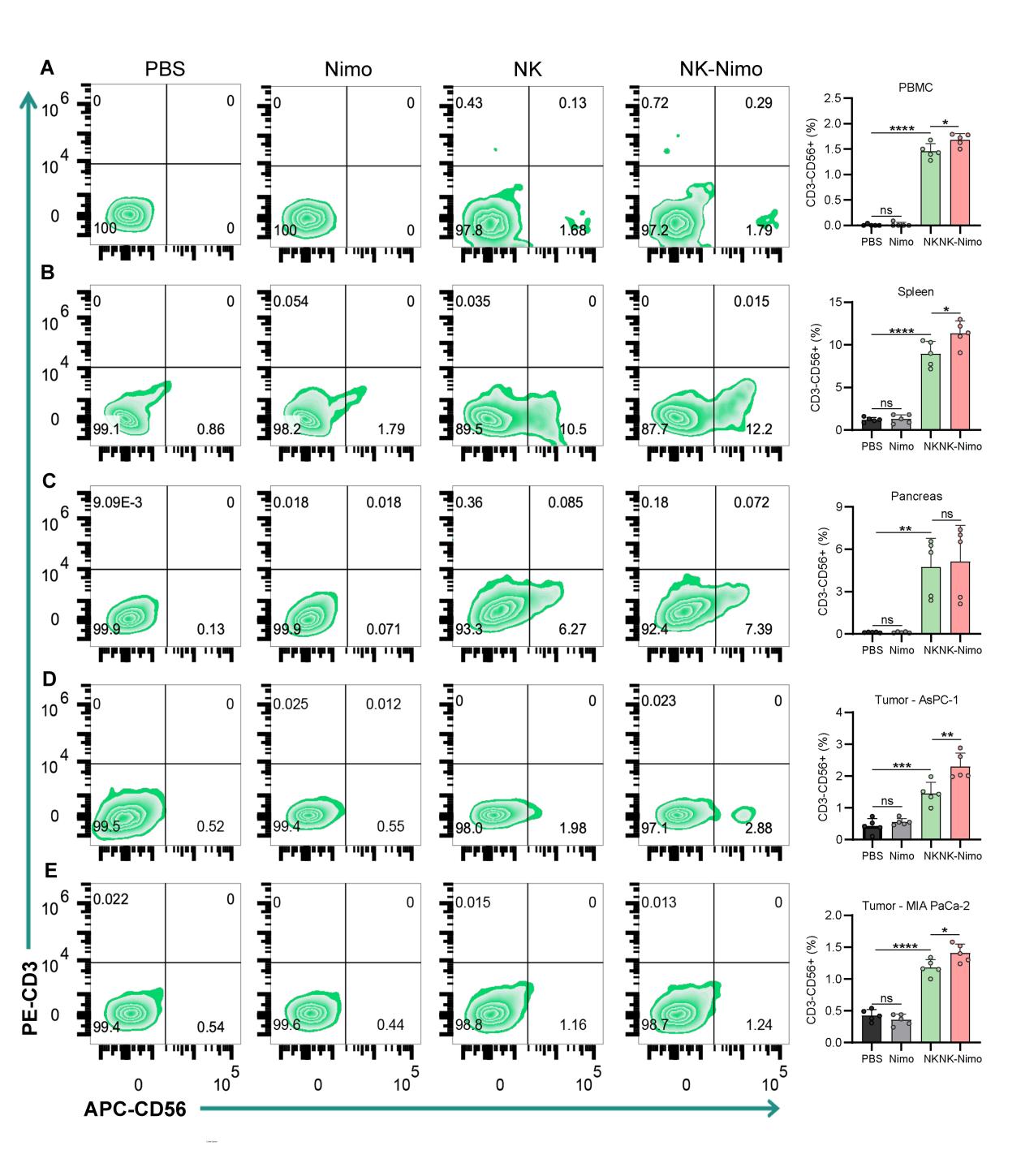


**Supplementary figure 12. NK cell activation across tissues in vivo.** Representative flow cytometry plots of activated NK cells (human NKG2D⁺/CD107a⁺) in (A) PBMCs and (B and C) bilateral tumors. Quadrant thresholds were independently calibrated for each tissue type using respective FMO and isotype controls to account for tissue-specific autofluorescence and signal-to-noise variations. Data were presented as mean + SD. Statistical significance was determined by one-way ANOVA with Tukey’s post-hoc test. *p < 0.05, **p < 0.01, ****p < 0.0001; ns, not significant. NK, natural killer; Nimo, nimotuzumab; PBMCs, Peripheral blood mononuclear cells.


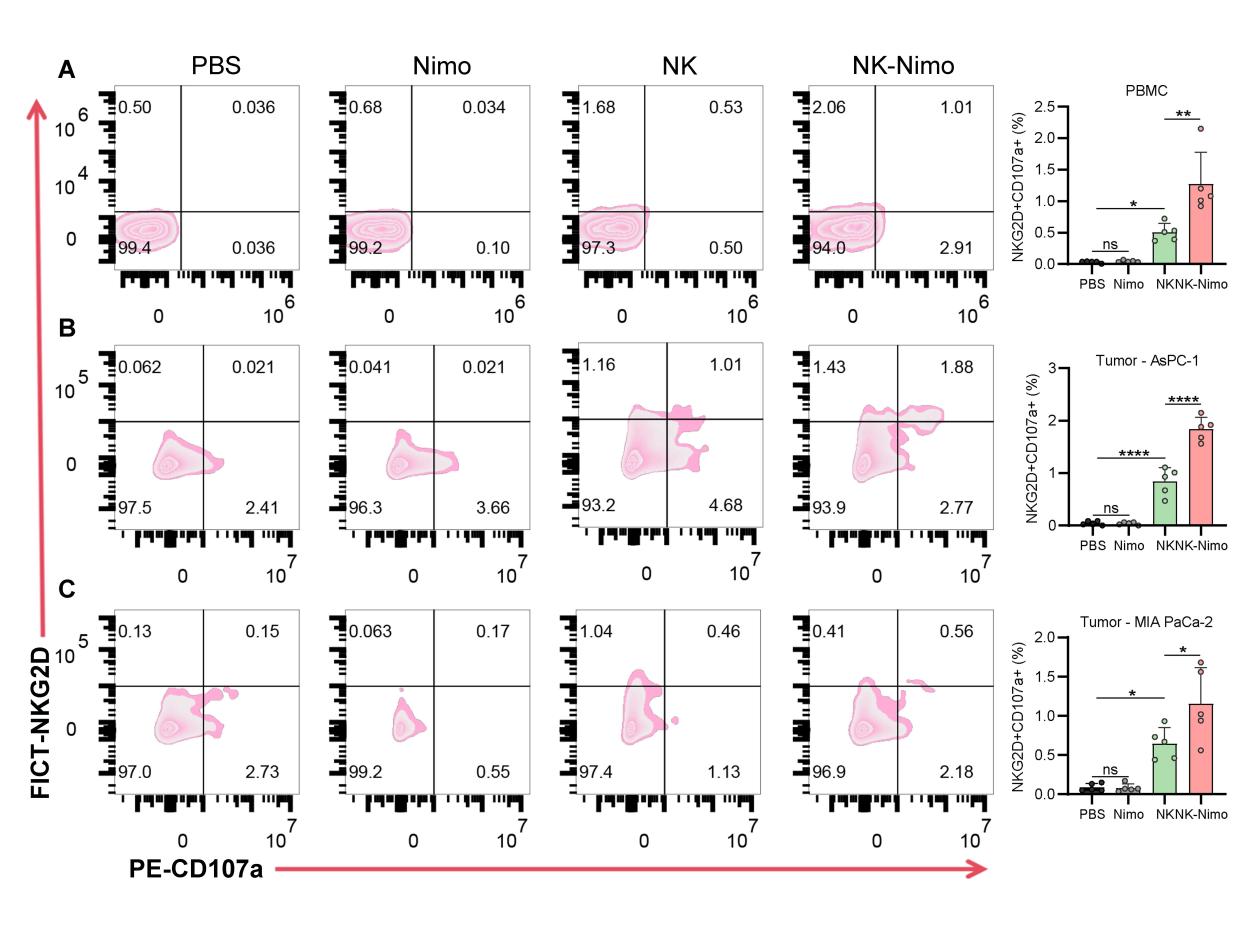


**Supplementary figure 13. Representative images of H&E-stained sections of major organs, including the heart, liver, spleen, lung, and kidney, harvested from mice at the therapeutic endpoint.**  Scale bar = 50 μm.


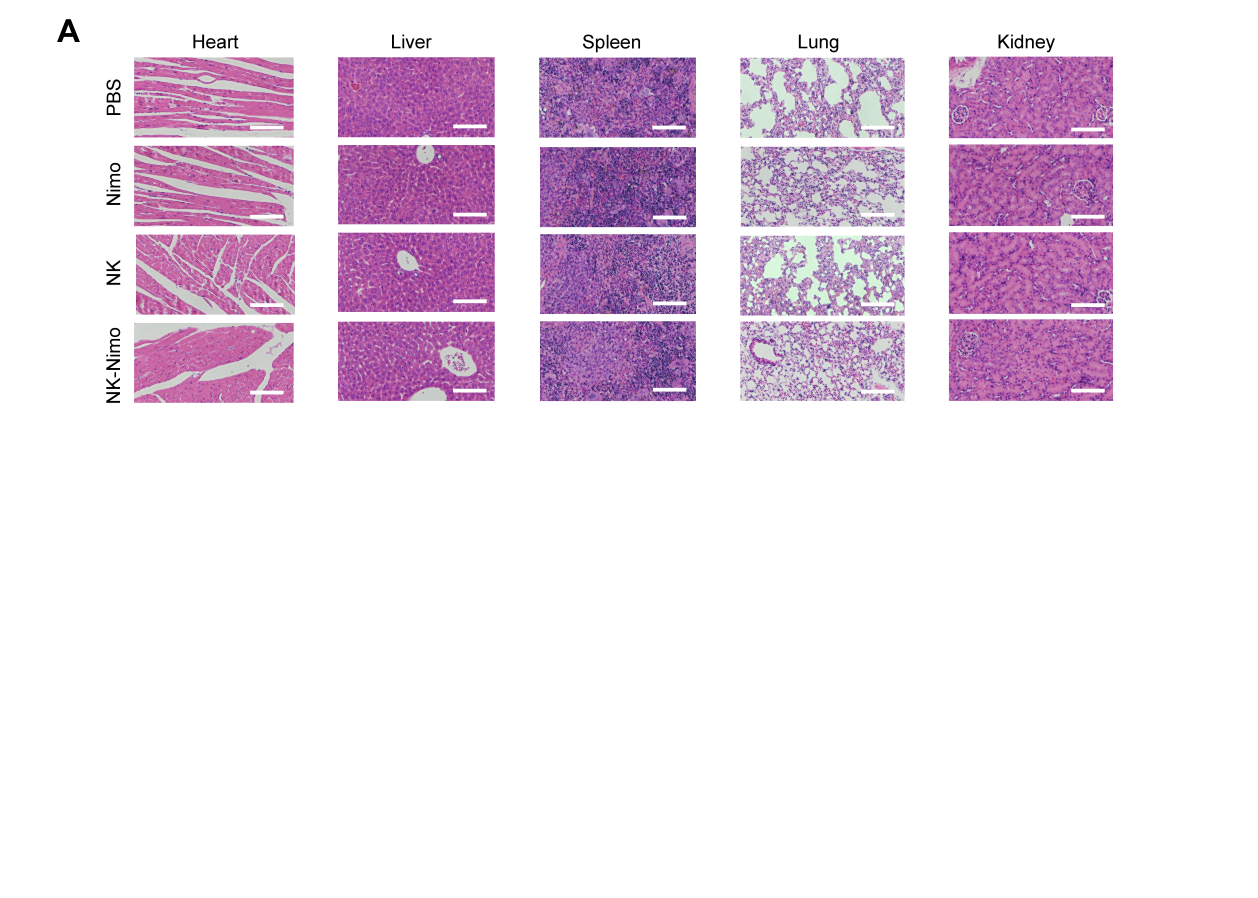

Supplement: Supplementary file 1 — Supporting File 1: mco270860‐sup‐0001‐SuppMat.docx [file MCO2-7-e70860-s001.docx]
